# Population attributable fraction of hypertension for dementia: global, regional, and national estimates for 186 countries

**DOI:** 10.1016/j.eclinm.2023.102012

**Published:** 2023-05-25

**Authors:** Martin D. Mulligan, Robert Murphy, Catriona Reddin, Conor Judge, John Ferguson, Alberto Alvarez-Iglesias, Emer R. McGrath, Martin J. O’Donnell

**Affiliations:** a School of Medicine, University of Galway, University Road, Galway, H91TK33, Ireland; b HRB Clinical Research Facility, University of Galway, University Road, Galway, H91TK33, Ireland

**Keywords:** Dementia, Alzheimer’s, Hypertension, PAF, Epidemiology

## Abstract

**Background:**

Quantifying the proportion of dementia attributable to highly prevalent modifiable risk factors, such as hypertension, is important in informing effective dementia prevention strategies. We aim to quantify the population attributable fraction (PAF) of hypertension for dementia (the proportion of dementia cases that would not occur if hypertension was eliminated) at global, regional, and national levels.

**Methods:**

In this study, we searched international and governmental websites for global, regional, and national data reporting population hypertension (according to 10-year age categories) and dementia prevalence. MEDLINE was searched for studies reporting the risk of dementia from age at hypertension diagnosis from database inception to December 31, 2022. Longitudinal observational studies with >500 participants reporting hazard ratios by age at hypertension diagnosis for risk of future all-cause dementia were eligible for inclusion. Studies excluded had cross-sectional methodology, specific vascular dementia or ‘cognitive impairment’ outcomes, and no age-specific metrics of association reported. The PAF of hypertension for dementia was calculated globally and for each country and region worldwide.

**Findings:**

Data from the Global Burden of Disease, United Nations Population Prospectus, NCD Risk Factor Collaboration, UK Biobank, and Atherosclerosis Risk in Communities Study were obtained. 186 countries reported dementia and hypertension prevalence data. The global PAF of hypertension for dementia was 15.8% [95% Credible Interval (CI), 8.8%–22.7%]. Latin America and the Caribbean (18.0% [95% CI, 9.4%–26.6%]), and Europe (17.2% [95% CI, 9.6%–24.7%]) had the highest PAF of hypertension for dementia. Hypertension diagnosed between the ages of 30–44 had the highest age-specific global attributable fraction for dementia (8.4% [95% CI, 3.4%–13.5%]), followed by ages 45–54 (2.92% [ 95% CI, 0.96%–4.88%]), 55–64 (2.59% [95% CI, 1.15%–4.03%]) and 65–74 (1.82% [95% CI, −2.31%–5.96%]).

**Interpretation:**

The population attributable risk of hypertension for dementia is 15.8%, suggesting that optimal detection and treatment, particularly at midlife, has the potential to markedly reduce the global burden of dementia.

**Funding:**

10.13039/100010269Wellcome Trust; Health Research Board of Ireland; 10.13039/100000957Alzheimer's Association.

**Research in context**

**Evidence before this study**

We did a literature review of the English language scientific literature in PubMed published in PubMed up to December 31, 2022 using the search terms ‘population attributable fraction’, ‘hypertension’ and ‘dementia’, and their synonyms. To date, the only international study to report the PAF of hypertension for dementia is the 10/66 Dementia Research study, which reports the PAF for eight countries worldwide. In addition, individual country estimates exist for the US, China, India, New Zealand, Brazil and Chile. The 10/66 study, which based estimates on prior history of diagnosed hypertension and may have underestimated the prevalence of hypertension. An alternate approach to estimate the PAF of hypertension for dementia is to apply previously published best estimates of association between hypertension and dementia to prevalence estimates of the two variables.

**Added value of this study**

This study provides estimates for the PAF of hypertension for dementia at global, regional and national levels for 186 countries worldwide and informs public health strategies for dementia prevention.

**Implications of all the available evidence**

These estimates can help inform public health policy at a global and national level and highlight the significant potential impact of hypertension modification, particularly midlife hypertension, in preventing dementia cases worldwide.

## Introduction

Dementia is a major contributor to years lived with disability, with an estimated 57 million individuals living with dementia globally and an estimated future increase in prevalence.^1^, ^2^, ^3^ Hypertension, particularly at earlier age of diagnosis in midlife, is associated with incident dementia.^4^, ^5^, ^6^ Rates of hypertension detection (46.5%) and control (32.5%) are suboptimal globally, with greater deficiencies in diagnosis and treatment in low-income countries relative to high income countries.^7^ Blood pressure lowering is associated with a modest reduction in the risk of dementia (∼7% relative risk reduction), based on a recent meta-analysis of randomized controlled blood pressure trials.^8^ Given the high prevalence of hypertension and dementia, however, even small risk reductions may translate into large numbers of dementia cases prevented in the population.^9^

Global strategies to reduce the burden of dementia need to be informed by the expected benefits from implementing various public health interventions. Population Attributable Fractions (PAFs) estimate the proportion of disease cases that would not occur if the risk factor was eliminated.^10^ To date, estimates for the PAF related to hypertension are derived from the 10/66 study, which based estimates on prior history of diagnosed hypertension and may have underestimated the prevalence of hypertension.^11^ An alternate approach to estimate the PAF of hypertension for dementia is to apply previously published best estimates of association between hypertension and dementia to prevalence estimates of the two variables. Given that numerous epidemiology studies have reported differences in prevalence rates according to age group,^5^^,^^7^ this approach also requires estimating the magnitude of association of hypertension with dementia in different age groups. In this study, we sought to estimate the PAF of hypertension for dementia at global, regional and national levels and provide updated estimates to inform public health strategies for dementia prevention.

## Methods

### Search strategy and selection criteria

**Fig. 1:**
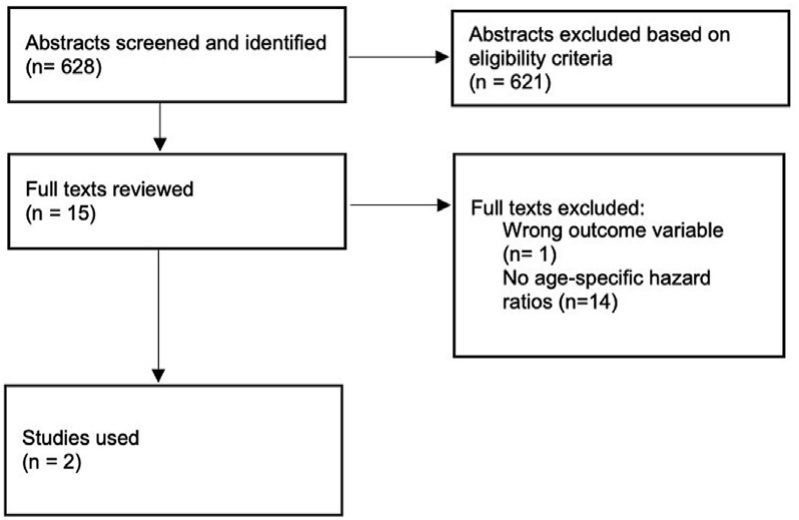
PRISMA flow diagram.

For estimates of population-level prevalence of hypertension and dementia, we searched international and governmental websites for high quality publicly available databases (Supplementary Methods 1). Adhering to MOOSE reporting guidelines, we searched MEDLINE (database inception to December 31, 2022) for longitudinal observational studies reporting age-specific associations between hypertension and dementia.^12^ Further details are included in the Supplementary Appendix, Supplementary Methods 2. Following completion of the search strategy, two investigators (MM and RM) independently screened titles and abstracts using Rayyan web application.^13^ Full texts of any potentially eligible articles were independently reviewed by two reviewers (MM and RM) and eligibility criteria (as described below) applied. 628 studies reporting an association between hypertension and dementia were identified. Following abstract screening based on the eligibility criteria, 15 studies underwent full text review (Fig. 1). In cases of disagreement, consensus was reached following formal discussion with a senior reviewer (MOD).

Observational studies relating age at hypertension diagnosis to risk of future all-cause dementia, and which included >500 participants from a community-based setting, with available longitudinal follow-up data (given the potentially long lag phase between hypertension and dementia clinical presentation^14^) and which reported hazard ratios by age group were eligible for inclusion. Studies excluded were composed of a specific non-community dwelling cohort of patients (for example, inpatient cohort), had cross-sectional methodology, reported exclusively for vascular dementia association, no age-specific metrics of association were reported or ‘cognitive impairment’ was reported as the primary outcome variable (which may refer to a collection of heterogenous conditions referring to mild cognitive impairment (a pre-dementia state with preserved independence which may or may not progress to dementia) or impairment with a reversible aetiology^14^).

### Data analysis

Population data were extracted at global, regional (6 United Nations geoscheme regions–Africa, Asia, Europe, Latin America and the Caribbean, Northern America, and Oceania) and national levels^15^ from United Nations World Population Prospectus 2019.^16^ Dementia prevalence data were obtained from the Global Burden of Disease (2019 estimates).^1^ Global hypertension prevalence data, subdivided by 10-year age groups, were obtained from the NCD Risk Factor Collaboration (NCD-Risc) dataset for people aged 30–79 years in 200 countries and territories between 1990 and 2019.^9^ Hypertension was defined as systolic blood pressure of 140 mmHg or greater, diastolic blood pressure of 90 mmHg or greater, or taking anti-hypertensive medication.^17^

Multivariable-adjusted hazard ratios for dementia based on age at diagnosis of hypertension were obtained and extracted from UK Biobank data, which provides age-specific hazard ratios based on age at hypertension diagnosis (i.e. <35 years old, 35–44 years, 45–54 years and 55–64 years).^6^ For the age 65–75 group, we used the ARIC study which reported hazard ratios in individuals with late-life hypertension only (i.e. confirmed mid-life normotension).^4^^,^^6^ We excluded the subpopulation of individuals with a new diagnosis of hypertension over age 75 years, due to significant heterogeneity in estimates across studies, possibly due to the competing effects of lower blood pressure.^5^^,^^18^, ^19^, ^20^, ^21^

The PAF is the proportion of all cases in the population (exposed and unexposed) that may be attributed to the exposure (PAF: [prevalence in total population—prevalence in unexposed]/prevalence in total population).^22^

To get precise estimates of PAF within country, c, we estimated separately for males and females (g∈{m,f}) using the following formula:

(1) $$P\hat{A}F_{c,g}=P\hat{A}F_{<35,c,g}+P\hat{A}F_{35-44,c,g}+P\hat{A}F_{45-54,c,g}+P\hat{A}F_{55-64,c,g}+P\hat{A}F_{>65,c,g}$$

where

(2) $$P\hat{A}F_{a,c,g}=\frac{{\hat{\pi }}_{a,c,g}\left(R{\hat{R}}_{a}-1\right)}{1+{\hat{\pi }}_{a,c,g}\left(R{\hat{R}}_{a}-1\right)}$$

is the estimated PAF due to incident hypertension in age group a. $\hat{\pi_{a,c,g}}$ is the estimated prevalence of incident hypertension in age group a, within a specific country and gender, and $\hat{RR_{a}}$ is the estimated relative risk of dementia due to incident hypertension at age group *a*, assumed constant across country and gender. The following is a worked example with further details about how the PAF and probabilities of incident hypertension in (2) are calculated described in Supplementary Methods 3.

To calculate incident hypertension in the 45–54 age group in Ireland, we subtract the prevalence of hypertension in the 50–54 age group from the prevalence of hypertension in the 40–44 age group (36.7%–21.6% = 15.1%). The relative risk of dementia in the 45–54 age group is 1.19.6

PAF of hypertension for dementia in the 45–54 age group in Ireland = 100 × (15.1/100)∗(1.19–1)/(1+(15.1/100)∗(1.19–1)) = 2.79%.

Number of cases that might be prevented by eliminating incident hypertension between 45 and 54 (PAF × number of dementia cases): 81,923 × 2.79 = 2285 cases (Further detail regarding this calculation available in Supplementary Spreadsheet 1).

Relative risks for dementia due to incident hypertension in each age group were approximated with estimated hazard ratios from UK Biobank data.^6^ Hazard ratios for dementia based on age at hypertension diagnosis from the UK Biobank and ARIC studies are summarised in Table 1. Statistical analyses were performed in R statistical software, version 4.1.3.

**Table 1:** Summary of hypertension prevalence, hazard ratios of hypertension for dementia based on age at diagnosis and population attributable fractions of hypertension for dementia by age group.

Region	30–44		45–54		55–64		65–74		Total	
HR	1.61 (1.31–1.99)^a^		1.19 (1.06–1.33)		1.17 (1.08–1.27)		1.16 (0.84–1.61)			
	HTN (%)	PAF (95% CI)	HTN (%)	PAF (95% CI)	HTN (%)	PAF (95% CI)	HTN (%)	PAF (95% CI)	HTN (%)	PAF (95% CI)
Global	18.5	8.44 (3.36–13.52)	33.6	2.92 (0.96–4.88)	47.8	2.59 (1.15–4.03)	59.2	1.82 (−2.31 to 5.96)	32.8	15.77 (8.80–22.74)
Africa	21.2	10.25 (3.28–17.23)	37.6	2.82 (0.93–4.71)	51.0	2.16 (0.95–3.36)	60.0	1.23 (−1.48 to 3.93)	32.2	16.46 (8.66–24.25)
Asia	18.0	8.27 (3.29–13.25)	32.3	2.80 (0.86–4.74)	45.4	2.39 (0.94–3.85)	55.3	1.63 (−2.20 to 5.45)	31.2	15.09 (8.48–21.70)
Europe	16.6	8.36 (3.50–13.21)	35.3	3.34 (1.10–5.59)	54.1	3.17 (1.42–4.91)	69.3	2.30 (−2.79 to 7.39)	39.2	17.17 (9.60–24.73)
L.A. and the Caribb.	22.0	10.48 (3.41–17.56)	39.4	3.05 (0.91–5.19)	55.1	2.57 (1.05–4.09)	67.6	1.87 (−2.37 to 6.12)	37.6	17.97 (9.39–26.56)
N.A.	14.0	7.28 (2.31–12.26)	30.3	2.86 (0.65–5.07)	47.3	2.91 (0.97–4.84)	62.2	2.35 (−3.16 to 7.86)	34.1	15.39 (7.69–23.09)
Oceania	14.2	7.01 (2.20–11.82)	29.2	2.72 (0.61–4.83)	45.3	2.78 (0.91–4.66)	60.0	2.30 (−3.09 to 7.86)	31.0	14.81 (7.32–22.30)

HR: Hazard Ratio; HTN: hypertension prevalence; L.A. and the Caribb.: Latin America and the Caribbean; N.A.: North America.

a The analysis combined the UK Biobank estimates for 30–34 (1.24% [95% CI, 0.91%–1.70%]) and 35–44 (1.61% [(95% CI, 1.31%–1.99%)]).

### Role of the funding source

The funders of the study had no role in study design, data collection, data analysis, data interpretation, or writing of the report. MM, RM, CR, CJ, JF, AAI, EMcG, MOD had access to the dataset. All authors were consulted and accept final responsibility for the decision to submit for publication.

## Results

186 countries reporting data on both dementia and hypertension prevalence were included in our analysis.

Global mean hypertension prevalence (age 30–79 years) was 33.7% (95% CI, 31.4%–36.1%). Prevalence of hypertension from NCD-Risc by region are described in the Supplementary material for 186 countries (Supplementary Table S2 and Supplementary Fig. S2).

Worldwide, 57.4 million (95% CI, 50.3 million–65.1 million) cases of dementia were recorded in 2019. Among the 186 countries, dementia cases ranged from 199 cases (95% CI, 166–220) in Samoa to 15.3 million (95% CI, 13.4 million–17.6 million) in China. Dementia prevalence is further described in Table 2 and Supplementary Fig. S3.^1^

**Table 2:** Total number of dementia cases globally and by region with number of dementia cases attributable to hypertension (based on age at hypertension diagnosis) by region and age group.

Region	Total dementia cases	Cases attributable to HTN overall (95% CI)	30–44 (95% CI)	45–54 (95% CI)	55–64 (95% CI)	65–74 (95% CI)
**Global**	60,335,304	9,513,747 (5,309,997–13,717,497)	5,090,519 (2,025,301–8,155,737)	1,763,310 (580,858–2,945,761)	1,560,349 (691,889–2 428,809)	1,099,569 (−1,393,872 to 3,593,009)
**Africa**	2,874,718	473,088 (249,080–697,097)	294,800 (94,269–495,332)	81,020 (26,667–135,373)	61,951 (27,260–96,642)	35,317 (−42,452 to 113,085)
**Asia**	35,862,304	5,411,176 (3,039,877–7,782,476)	2,964,471 (1,178,294–4,750,647)	1,004,912 (309,645–1,700,180)	858,212 (336,498–1,379,927)	583,581 (−787,583 to 1,954,745)
**Europe**	10,831,805	1,859,398 (1,040,363–2,678,433)	905,106 (379,059–1,431,152)	361,902 (118,697–605,107)	342,925 (153,951–531,899)	249,465 (−302,041 to 800,971)
**L.A. and the Caribb.**	4,476,200	804,455 (420,189–1,188,720)	469,263 (152,690–785,836)	136,351 (40,523–232,179)	115,003 (47,074–182,932)	83,837 (−106,238 to 273,913)
**Northern America**	5,856,257	901,350 (450,281–1,352,420)	426,457 (135,151–717,763)	167,323 (37,838–296,808)	170,170 (56,764–283,577)	137,400 (−185,229 to 460,029)
**Oceania**	434,020	64,279 (31,790–96,769)	30,422 (9540–51,305)	11,801 (2644–20,958)	12,087 (3933–20,242)	9969 (−13,407 to 33,344)

**Fig. 2:**
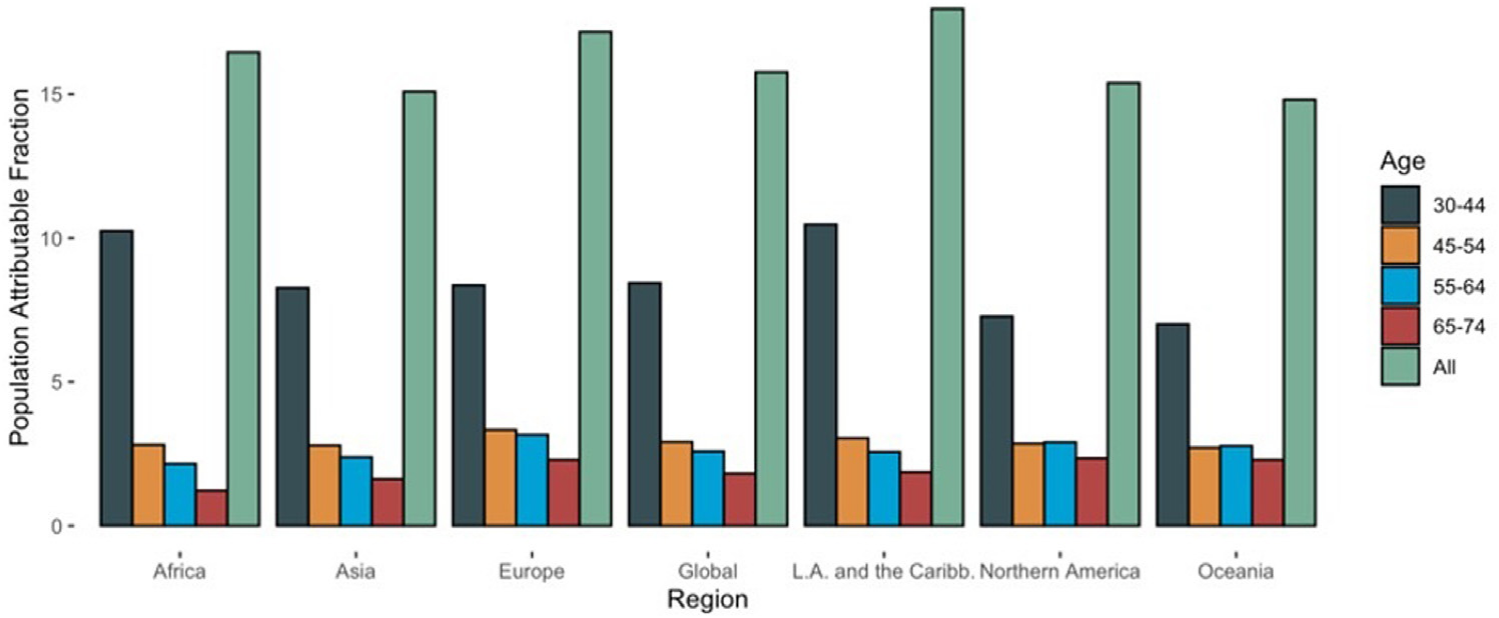
Population attributable fractions of hypertension for dementia (based on age at hypertension diagnosis) by region.

**Fig. 3:**
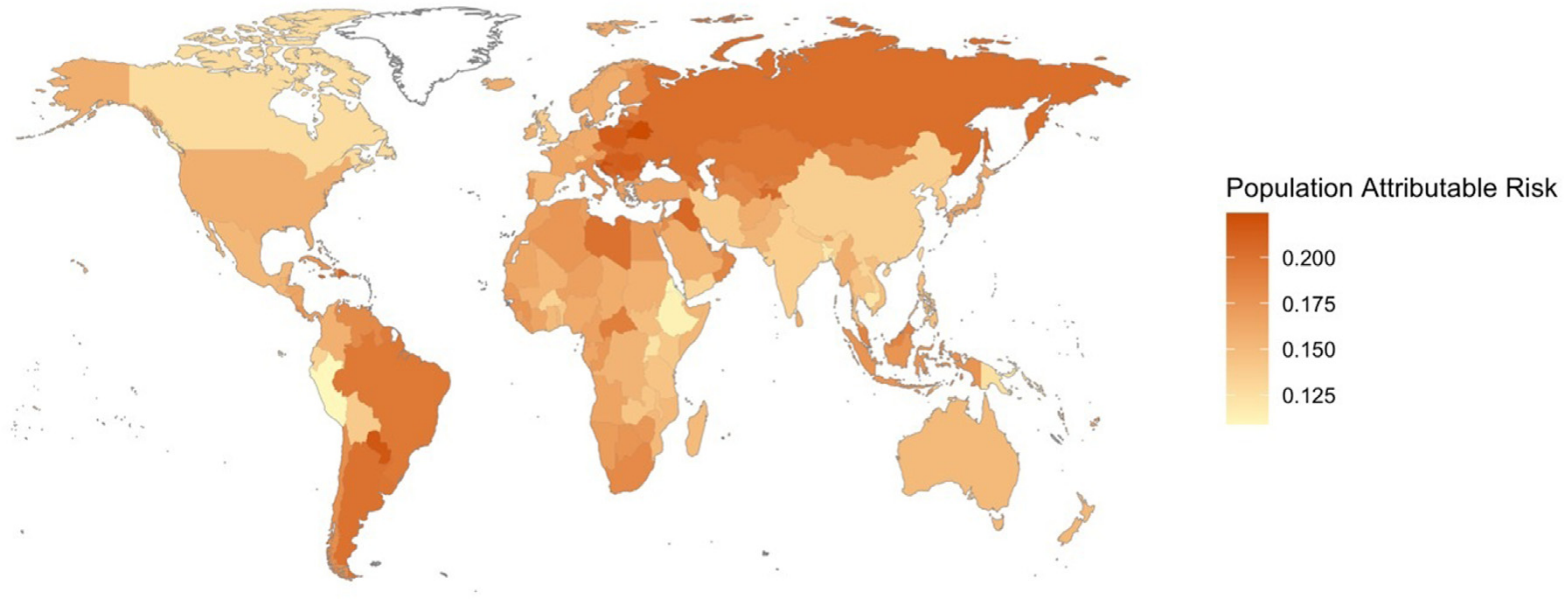
World map of population attributable risk of hypertension for dementia by country.

The global population Attributable Fraction of hypertension for dementia was estimated to be 15.8% (95% CI, 8.8%–22.7%), ranging from 14.8% (95% CI, 7.3%–22.3%]) in Oceania to 18.0% (95% CI, 9.4%–26.6%) in Latin America and the Caribbean (Table 1, Fig. 2). Within age groups, the PAF was highest (8.4% [95% CI, 3.4%–13.5%]) in the 30–44 age group, 2.9% (95% CI, 1%–4.9%) in the 45–54 age group, 2.6% (95% CI, 1.2%–4%) in the 55–64 age group and lowest (1.8% [95% CI, −2.3%–6%]) in the 65–74 age group (Table 1, Fig. 2). The PAF for men was 16% (95% CI, 84%–23.6%) and 15.7% (8.7%–22.6%) for women with estimates by region in Supplementary Table S3 and Supplementary Fig. S1. Among individual countries, the PAF for hypertension ranged from Eritrea (11.1% [95% CI, 3.6%–18.6%]) to (22.5% [95% CI, 12.5%–32.4%]) in Belarus (Fig. 3, Supplementary Tables S4 and S5). Among men, the PAF ranged from 9.8% [95% CI, 0.4%–19.2%]) in Eritrea to 22.6% [95% CI, 12.0%–33.3%]) in Czech Republic. Among women, the PAF ranged from 10.8% [95% CI, 2.7%–19.0%]) in Switzerland to 22.8% [95% CI, 12.8%–32.8%]) in Belarus (Supplementary Table S4).

In 2019, an estimated 9.5 million (95% CI, 5.3 million–13.7 million) cases worldwide were attributable to hypertension, ranging from 1.9 million (95% CI, 1.0 million–2.6 million) in Europe to 5.4 million (95% CI, 3.0 million–7.8 million) in Asia (Supplementary Table S1). Diagnosis of hypertension between the ages of 30–44 years accounted for 5 million (95% CI, 2.0 million–8.2 million) dementia cases globally, of which 3 million (95% CI, 1.2 million–4.8 million) cases were in Asia and 900,000 (95% CI, 379,000–1.4 million) cases in Europe. The regional order of frequency of attributable cases was consistent across age groups, with Asia, Europe, and North America having the highest numbers, followed by Latin America and the Caribbean, Africa, and Oceania (Table 2 and Supplementary Fig. S4). The number of dementia cases attributable to hypertension by sex are summarised in Supplementary Fig. S1. The number of dementia cases per country attributable to hypertension total and by age range are described in Supplementary Table S6.

## Discussion

The Global Population Attributable Fraction of hypertension for dementia is 15.8% (95% CI, 8.8%–22.7%), with the highest attributable fraction in those developing hypertension at age 30–44 years (PAF 8.4% [95% CI, 3.4%–13.5%]). In 2019, we estimate that 9.5 million cases of dementia were attributable to hypertension globally. Our findings, in the context of a recent meta-analysis reporting a significant reduction in dementia risk with anti-hypertensive therapy, suggest that a large proportion of dementia cases may be prevented with optimal detection and treatment of hypertension globally.^8^ Moreover, identification and treatment of hypertension in early and mid-adult life is of particular importance in reducing the global burden of dementia. The World Health Organisation’s global action plan for dementia recommends treatment of midlife hypertension to reduce the risk of dementia; the potential impact of these recommendations is highlighted by this study.^23^

The only international study to report the PAF of hypertension for dementia is the 10/66 Dementia Research study, which included participants from India, China and six Latin America countries (Cuba, Dominican Republic, Mexico, Peru, Puerto Rico, and Venezuela.^11^ In that study, the estimated PAF for hypertension was 3.9%, which is considerably lower than the estimate derived in our study. However, in that study, the prevalence of hypertension was based on a prior history of hypertension, which would have underestimated the frequency of hypertension. Among individual country studies, modelled estimates in China have reported similar estimates of PAF, 18.6% and 22.1% (compared to our more conservative estimate of 13.6%), however, a relative risk (RR) of hypertension for dementia was uniformly applied to all ages instead of age-specific RR (as in our study).^11^^,^^24^

In the United States (US), previous modelling studies estimated PAFs of 12 modifiable risk factors for dementia, of which, the greatest attributable fraction was for hypertension (20.2%).^25^ Our lower estimate of 15.7% may be explained by the hazard ratios used in our analysis being more sensitive to age-specific risk (compared to the uniform RR of 1.61 applied to all ages in the previous study).^25^ Modelled estimates from other countries have been reasonably consistent with our estimates, including India (reported PAF of 10.4% versus 14.0% in our study), New Zealand (16.7% compared to our estimate of 15%), Chile (12.5%; smaller than our estimate of 18.0%, possibly due to excluding the 30–44 age group with the highest attributable fraction in our study) and Brazil (23.8% compared to our estimate of 19.6%).^11^^,^^26^, ^27^, ^28^

Treatment of hypertension, with antihypertensive therapy, is associated with a 7% relative reduction in the odds of dementia, based on a meta-analysis of 12 randomised controlled trials and 92,135 participants.^8^ Another meta-analysis reported a 12% relative reduction in populations achieving a greater than 10 mmHg blood pressure.^29^ Therefore, the impact fraction related to identification and control of hypertension is expected to be lower than the reported PAF related to hypertension, as residual risk will remain related to hypertension. Some of that residual risk is expected to be related to periods of undiagnosed hypertension, particularly among those in younger age groups, for which we report the largest PAF. While the relative risk reduction of associated with antihypertensive therapy is modest, this may result in a substantial public health benefit worldwide, given the high prevalence of both hypertension and dementia.

Given that hypertension is largely asymptomatic, midlife hypertension is often underdiagnosed and undertreated worldwide despite having a significant effect on dementia prevalence.^5^ The global prevalence of hypertension is 32.8%, with the prevalence in the 30–44 age group (those with the longest hypertension exposure and highest dementia risk) being 18.5%.^9^ Of 142,000 study participants who had hypertension across 17 countries worldwide as part of the PURE (Prospective Urban Rural Epidemiology) study, only 46.5% (95% CI, 46.1%–46.9%) were aware of the diagnosis, with only 32.5% of those diagnosed (95% CI, 31.9%–33.1%) achieving blood pressure control. The scope for improvement in blood pressure detection and control was highest in low income countries (p < .001).^7^ A study examining the trend of hypertension prevalence, awareness, and treatment between 1990 and 2019 corroborated the above findings with the largest improvements in awareness and treatment in high- and upper-middle-income countries with little change in most countries in Sub-Saharan Africa and Oceania.^30^ The contributions of hypertension to dementia can be reduced by enabling and promoting access to healthcare both in low income countries, where just 27% of poor households (defined as being in the lowest two income quintiles) report treatment for all chronic conditions, and in high income countries, where only 51% of poor households receive treatment for all chronic conditions.^31^ Our study details the practical implications of evidence from the UK Biobank and ARIC where the duration of exposure to hypertension increases the odds of dementia and highlights at risk populations that can be targeted for early hypertension diagnosis and treatment.^4^^,^^6^

There are several mechanisms by which hypertension may increase an individuals risk of dementia, including through a direct effect on brain structure and microvasculature (such as stroke (and covert stroke)), and indirect effect through increasing risk of other conditions such as heart failure, which also incur increased risk of cognitive impairment and dementia.^32^

This study has several limitations. First, the estimates of dementia prevalence from the GBD study are based on heterogenous data given the absence of standardised methodologies for dementia measurement in observational studies across regions.^33^ We report the risk of all-cause dementia as our primary outcome, due to heterogeneity of dementia measurement across studies and the clinical overlap between dementia types. While this may regarded as a potential limitation due to recommended diagnostic criteria from the WHO International Classification of Diseases (ICD) 11 requiring clinicians to specify subtypes including Alzheimer’s and Vascular dementia,^34^ we argue that an all-cause dementia outcome variable reflects the clinical overlap frequently present between these subgroups.^35^ The association of vascular disease with dementia prevalence is well described; of the 12 modifiable risk factors identified by the Lancet Commission, many are vascular risk factors including hypertension, smoking, midlife obesity, diabetes and physical inactivity.^36^ Those with the APOE e4 gene have an increased odds of MRI markers of cerebrovascular disease and clinical outcomes of hypercholesterolaemia and ischaemic heart disease.^37^^,^^38^ Second, hypertension data used relied on previous diagnosis and treatment of hypertension possibly leading to measurement error. However, validation studies show that recall of hypertension diagnosis and medication use has good agreement with previous documented medical history.^39^, ^40^, ^41^ Third, our PAF is based on hypertension defined as >140 mmHg/90 mmHg. However, systolic blood pressure in the 130–140 mmHg range is associated with an increase in prevalence of cardiovascular diseases compared to those with lower blood pressure.^42^ No study has determined the precise blood pressure cut-off above which dementia risk is increased, however, it is possible that our PAF values may be underestimations. Fourth, there has been no large prospective cohort international study quantifying the relationship of hypertension and dementia. Our hazard ratios obtained from United Kingdom and United States populations have been applied to regions with differing nutritional status, cultural and environmental influences which may affect hypertension’s interaction with dementia.

This is the first study that provides global, regional, and country estimations for 186 countries, describing global estimates stratified by age of hypertension diagnosis. With the global expenditure annual expenditure on dementia morbidity estimated at US $2.8 trillion, our reported figures demonstrate a large population worldwide that can be targeted through cost-effective public health programs and reduce long-term healthcare system dependence.^43^ For the primary outcome in this study, we selected an all-cause dementia variable. Epidemiological and pathological studies indicate that it is possible that dementia is a continuum of pathologies with clinical diagnoses of Alzheimer’s and vascular dementia at ends of a spectrum.^35^ The composite variable of all-cause dementia demonstrates the large clinical cohort that can be positively affected through effective public health intervention.

In conclusion, hypertension is associated with a 15.8% population attributable risk for dementia, suggesting that optimal detection and treatment has the potential to markedly reduce the global burden of dementia.

## Contributors

MDM was responsible for formal analysis, data curation, visualisation, writing–original draft, and writing–review & editing. RM was responsible for data curation, investigation, and methodology. CR was responsible for data curation, investigation, and methodology. CJ was responsible for data curation, investigation, methodology, supervision, and writing–review & editing. JF was responsible for formal analysis, data curation, and writing–review & editing. AAI was responsible for formal analysis. ERMG was responsible for supervision and writing–review & editing. MJOD was responsible for conceptualisation, investigation, methodology, supervision, and writing–review & editing. MM, RM, CR, and JF have accessed and verified the underlying data. All authors read and approved the final version of the manuscript.

## Data sharing statement

All data obtained is publicly available as described and available from Global Burden of Disease, NCD Risk Factor Collaboration, United Nations website, UK Biobank and ARIC published studies.

## Declaration of interests

CR was supported by the Irish Clinical Academic Training (ICAT) Programme, the Wellcome Trust and the Health Research Board (grant number 203930/B/16/Z). JF received Grant EIA-2017-017 from the Health Research Board Ireland from 1/10/2017 to 1/10/2021 (this period didn’t coincide with intellectual work done on the manuscript). ERMG is in receipt of funding from the Health Research Board of Ireland (CSF-2020-011) and the Alzheimer’s Association (AACSF-18-566570). MJOD received Health Research Board DIFA Award (for randomised controlled trial in Atrial fibrillation screening). The funding source had no role in the design and conduct of the study or the publication decision.

## Appendix ASupplementary data

Supplementary Spreadsheet 1
