# Supplementary material for: Population attributable fraction of hypertension for dementia: global, regional, and national estimates for 186 countries
Source: eClinicalMedicine. 2023 May 25;60:102012. doi: 10.1016/j.eclinm.2023.102012 (PMC10227413; doi:10.1016/j.eclinm.2023.102012)

## **Supplementary Table 1 – Number of dementia cases attributable to hypertension (based on age at hypertension diagnosis) by region and age group.**

| **Region** | **30-44 (95% CI)** | **45-54 (95% CI)** | **55-64 (95% CI)** | **65-74 (95% CI)** | **Total (95% CI)** |
| --- | --- | --- | --- | --- | --- |
| **Global** | 5 090 519  (2 025 301 to 8 155 737) | 1 763 310  (580 858 to 2 945 761) | 1 560 349  (691 889 to 2 428 809) | 1 099 569  (-1 393 872 to 3 593 009) | 9 513 747  (5 309 997 to 13 717 497) |
| **Africa** | 294 800  (94 269 to 495 332) | 81 020  (26 667 to 135 373) | 61 951  (27 260 to 96 642) | 35 317  (-42 452 to 113 085) | 473 088  (249 080 to 697 097) |
| **Asia** | 2 964 471  (1 178 294 to 4 750 647) | 1 004 912  (309 645 to 1 700 180) | 858 212  (336 498 to 1 379 927) | 583 581  (-787 583 to 1 954 745) | 5 411 176  (3 039 877 to 7 782 476) |
| **Europe** | 905 106  (379 059 to 1 431 152) | 361 902  (118 697 to 605 107) | 342 925  (153 951 to 531 899) | 249 465  (-302 041 to 800 971) | 1 859 398  (1 040 363 to 2 678 433) |
| **L.A. and the Caribb.** | 469 263  (152 690 to 785 836) | 136 351  (40 523 to 232 179) | 115 003  (47 074 to 182 932) | 83 837  (-106 238 to 273 913) | 804 455  (420 189 to 1 188 720) |
| **Northern America** | 426 457  (135 151 to 717 763) | 167 323  (37 838 to 296 808) | 170 170  (56 764 to 283 577) | 137 400  (-185 229 to 460 029) | 901 350  (450 281 to 1 352 420) |
| **Oceania** | 30 422  (9 540 to 51 305) | 11 801  (2 644 to 20 958) | 12 087  (3 933 to 20 242) | 9 969  (-13 407 to 33 344) | 64 279  (31 790 to 96 769) |

**Supplementary Table 2– Dementia case numbers and percentage of population with hypertension by country (Data extracted from GBD and NCD-Risc)**^1,9^**.**

| Country | Dementia cases | Total prevalence of HTN (%) | 30-34  HTN  (%) | 35-39  HTN  (%) | 40-44  HTN  (%) | 45-49  HTN  (%) | 50-54  HTN  (%) | 55-59  HTN  (%) | 60-64  HTN  (%) | 65-69  HTN  (%) | 70-74  HTN  (%) |
| --- | --- | --- | --- | --- | --- | --- | --- | --- | --- | --- | --- |
| Afghanistan | 55 734 | 45.8 | 23.3 | 28.2 | 33.9 | 39.8 | 45.5 | 50.5 | 54.6 | 58.1 | 61.0 |
| Albania | 36 079 | 50.0 | 16.7 | 22.9 | 30.9 | 40.0 | 49.4 | 57.9 | 64.8 | 69.9 | 73.1 |
| Algeria | 250 509 | 45.0 | 12.4 | 17.4 | 24.4 | 33.2 | 42.7 | 51.8 | 59.6 | 65.8 | 70.2 |
| Angola | 46 598 | 45.1 | 19.0 | 24.3 | 30.7 | 37.7 | 44.7 | 50.9 | 56.0 | 60.1 | 63.0 |
| Antigua and Barbuda | 592 | 50.7 | 19.9 | 25.3 | 32.4 | 40.4 | 48.8 | 56.6 | 63.4 | 69.2 | 73.8 |
| Argentina | 412 268 | 55.7 | 24.2 | 30.3 | 37.8 | 46.1 | 54.4 | 62.0 | 68.5 | 73.8 | 78.1 |
| Armenia | 31 890 | 55.6 | 22.0 | 28.7 | 37.0 | 46.3 | 55.5 | 63.7 | 70.1 | 74.9 | 78.0 |
| Australia | 348 673 | 38.1 | 9.0 | 12.6 | 17.8 | 24.7 | 32.9 | 41.6 | 50.1 | 57.9 | 64.6 |
| Austria | 146 391 | 43.4 | 10.7 | 15.1 | 21.4 | 29.5 | 38.8 | 48.3 | 57.2 | 65.0 | 71.4 |
| Azerbaijan | 52 070 | 49.4 | 16.4 | 22.2 | 29.8 | 38.7 | 48.0 | 56.7 | 63.9 | 69.5 | 73.4 |
| Bahamas | 1 920 | 51.8 | 24.1 | 29.2 | 35.7 | 43.0 | 50.4 | 57.3 | 63.2 | 68.0 | 72.0 |
| Bahrain | 5 126 | 46.0 | 14.9 | 19.9 | 26.7 | 35.1 | 44.2 | 52.7 | 59.9 | 65.5 | 69.4 |
| Bangladesh | 570 899 | 33.2 | 15.9 | 19.5 | 23.7 | 28.3 | 32.8 | 36.7 | 40.1 | 42.8 | 45.2 |
| Barbados | 3 023 | 51.1 | 15.6 | 21.7 | 29.7 | 38.9 | 48.7 | 57.8 | 65.9 | 72.6 | 78.0 |
| Belarus | 137 286 | 58.5 | 18.9 | 27.0 | 37.1 | 48.5 | 59.5 | 68.9 | 76.0 | 80.9 | 83.8 |
| Belgium | 190 477 | 39.7 | 7.5 | 11.4 | 17.2 | 24.9 | 34.1 | 43.8 | 53.2 | 61.6 | 68.7 |
| Belize | 1 366 | 45.4 | 18.2 | 22.8 | 28.8 | 35.8 | 43.2 | 50.3 | 56.5 | 61.9 | 66.3 |
| Benin | 17 550 | 37.4 | 12.7 | 16.9 | 22.5 | 29.2 | 36.3 | 42.9 | 48.4 | 52.7 | 55.5 |
| Bhutan | 2 588 | 48.7 | 26.5 | 31.4 | 37.1 | 43.0 | 48.6 | 53.4 | 57.4 | 60.7 | 63.5 |
| Bolivia | 43 829 | 36.0 | 10.5 | 13.9 | 18.6 | 24.6 | 31.6 | 38.8 | 45.8 | 52.5 | 58.8 |
| Bosnia and Herzegovina | 48 081 | 54.1 | 15.1 | 22.0 | 31.1 | 41.9 | 53.1 | 63.2 | 71.4 | 77.4 | 81.5 |
| Botswana | 6 737 | 50.1 | 24.2 | 29.4 | 35.8 | 42.7 | 49.6 | 55.7 | 60.8 | 64.9 | 68.0 |
| Brazil | 1 849 981 | 53.3 | 21.7 | 27.4 | 34.6 | 42.9 | 51.4 | 59.3 | 66.2 | 72.1 | 76.9 |
| Brunei Darussalam | 1 574 | 54.3 | 21.1 | 28.2 | 36.7 | 45.6 | 54.3 | 61.8 | 67.9 | 72.7 | 76.2 |
| Bulgaria | 135 285 | 54.1 | 18.2 | 24.8 | 33.4 | 43.3 | 53.4 | 62.6 | 70.0 | 75.4 | 79.1 |
| Burkina Faso | 33 793 | 35.8 | 14.0 | 18.1 | 23.3 | 29.2 | 35.4 | 41.0 | 45.7 | 49.1 | 51.1 |
| Burundi | 14 791 | 40.5 | 16.8 | 20.7 | 25.9 | 32.1 | 38.7 | 45.0 | 50.6 | 55.2 | 58.8 |
| Cabo Verde | 2 546 | 51.5 | 19.9 | 26.1 | 33.9 | 42.6 | 51.3 | 59.0 | 65.2 | 69.8 | 73.0 |
| Cambodia | 55 230 | 31.4 | 9.9 | 13.5 | 18.2 | 23.8 | 29.8 | 35.5 | 40.5 | 44.7 | 47.8 |
| Cameroon | 41 181 | 43.9 | 15.4 | 20.8 | 27.6 | 35.3 | 43.1 | 50.3 | 56.2 | 60.8 | 63.9 |
| Canada | 587 364 | 31.1 | 4.1 | 6.6 | 10.6 | 16.4 | 23.9 | 32.5 | 41.5 | 50.4 | 58.7 |
| Central African Republic | 8 207 | 49.5 | 16.2 | 22.8 | 30.9 | 39.8 | 48.7 | 56.6 | 63.2 | 68.6 | 72.6 |
| Chad | 20 134 | 43.9 | 19.0 | 24.1 | 30.3 | 37.1 | 43.9 | 50.0 | 54.8 | 58.4 | 60.4 |
| Chile | 174 921 | 45.9 | 11.5 | 16.5 | 23.4 | 32.0 | 41.6 | 51.2 | 60.0 | 67.9 | 74.7 |
| China | 15 330 045 | 34.1 | 8.9 | 12.9 | 18.3 | 24.7 | 31.8 | 38.7 | 44.8 | 50.0 | 54.0 |
| Colombia | 369 422 | 39.8 | 9.9 | 13.8 | 19.5 | 26.8 | 35.2 | 43.8 | 52.1 | 59.5 | 66.0 |
| Comoros | 2 074 | 39.7 | 15.4 | 19.4 | 24.7 | 31.0 | 37.8 | 44.2 | 49.9 | 54.6 | 58.4 |
| Congo | 11 538 | 46.7 | 18.9 | 24.4 | 31.2 | 38.6 | 46.1 | 52.8 | 58.4 | 62.8 | 66.0 |
| Costa Rica | 32 637 | 46.6 | 14.5 | 19.7 | 26.5 | 34.6 | 43.3 | 51.8 | 59.5 | 66.3 | 72.3 |
| Cote d'Ivoire | 34 438 | 44.0 | 16.8 | 22.2 | 28.9 | 36.2 | 43.7 | 50.4 | 55.8 | 59.8 | 62.4 |
| Croatia | 83 429 | 58.2 | 18.7 | 26.3 | 35.9 | 46.9 | 57.8 | 67.4 | 75.1 | 80.9 | 85.0 |
| Cuba | 116 055 | 47.5 | 18.6 | 23.7 | 30.2 | 37.8 | 45.7 | 53.1 | 59.6 | 65.0 | 69.3 |
| Cyprus | 14 143 | 40.7 | 7.7 | 11.9 | 18.0 | 26.0 | 35.4 | 45.3 | 54.6 | 62.9 | 69.7 |
| Czech Republic | 192 748 | 51.5 | 13.1 | 19.7 | 28.5 | 38.9 | 49.9 | 60.0 | 68.5 | 74.9 | 79.5 |
| Denmark | 81 923 | 44.5 | 14.6 | 18.9 | 24.8 | 32.3 | 40.7 | 49.3 | 57.1 | 63.9 | 69.5 |
| Djibouti | 2 085 | 40.7 | 16.9 | 20.8 | 26.0 | 32.2 | 38.9 | 45.2 | 50.8 | 55.4 | 59.0 |
| Dominica | 51 735 | 55.6 | 24.5 | 30.5 | 37.9 | 46.2 | 54.5 | 62.0 | 68.4 | 73.6 | 77.7 |
| Dominican Republic | 153 708 | 57.2 | 24.7 | 31.3 | 39.2 | 47.9 | 56.5 | 64.1 | 70.5 | 75.6 | 79.7 |
| DR Congo | 87 769 | 40.9 | 15.0 | 19.8 | 25.9 | 32.7 | 39.7 | 46.3 | 51.8 | 56.3 | 59.7 |
| Ecuador | 305 675 | 34.6 | 10.0 | 13.3 | 17.8 | 23.6 | 30.3 | 37.2 | 44.0 | 50.4 | 56.6 |
| Egypt | 36 675 | 46.0 | 15.8 | 21.0 | 27.9 | 36.1 | 44.7 | 52.7 | 59.4 | 64.5 | 67.8 |
| El Salvador | 2 416 | 40.0 | 13.0 | 17.3 | 22.9 | 29.7 | 37.2 | 44.5 | 51.2 | 56.9 | 61.8 |
| Equatorial Guinea | 7 666 | 45.1 | 17.8 | 23.1 | 29.7 | 36.9 | 44.3 | 50.9 | 56.5 | 61.0 | 64.3 |
| Eritrea | 26 710 | 29.2 | 9.9 | 12.5 | 16.2 | 21.0 | 26.5 | 32.1 | 37.3 | 41.9 | 45.9 |
| Estonia | 174 023 | 47.3 | 17.4 | 23.1 | 30.3 | 38.7 | 47.4 | 55.2 | 61.5 | 65.6 | 67.4 |
| Ethiopia | 3 125 | 32.0 | 14.9 | 17.5 | 21.2 | 25.7 | 30.6 | 35.4 | 39.5 | 42.9 | 45.3 |
| Fiji | 97 549 | 46.2 | 17.1 | 22.2 | 28.7 | 36.4 | 44.4 | 51.9 | 58.4 | 63.8 | 68.0 |
| Finland | 1 203 439 | 45.3 | 11.4 | 16.5 | 23.5 | 32.1 | 41.8 | 51.4 | 60.0 | 67.1 | 72.5 |
| France | 5 796 | 39.9 | 5.1 | 9.0 | 15.1 | 23.5 | 33.7 | 44.5 | 54.8 | 63.9 | 71.5 |
| Gabon | 3 796 | 44.1 | 17.5 | 22.7 | 29.0 | 36.1 | 43.3 | 49.8 | 55.2 | 59.6 | 62.8 |
| Gambia | 46 291 | 44.1 | 17.2 | 22.6 | 29.2 | 36.5 | 43.8 | 50.4 | 55.7 | 59.7 | 62.2 |
| Georgia | 1 691 221 | 52.9 | 19.6 | 25.5 | 33.4 | 42.5 | 52.0 | 60.6 | 67.6 | 73.0 | 76.6 |
| Germany | 57 276 | 39.7 | 6.8 | 10.6 | 16.4 | 24.3 | 33.8 | 43.9 | 53.6 | 62.4 | 69.8 |
| Ghana | 206 366 | 39.8 | 14.9 | 19.8 | 26.0 | 33.0 | 39.9 | 46.2 | 51.2 | 54.6 | 56.4 |
| Greece | 1 058 | 41.7 | 7.3 | 11.5 | 17.7 | 26.0 | 35.8 | 46.2 | 56.0 | 64.9 | 72.6 |
| Grenada | 60 721 | 54.5 | 23.9 | 29.6 | 36.8 | 44.9 | 53.2 | 60.7 | 67.1 | 72.4 | 76.6 |
| Guatemala | 21 227 | 39.9 | 12.2 | 16.3 | 21.9 | 28.9 | 36.6 | 44.4 | 51.4 | 57.6 | 62.7 |
| Guinea | 2 271 | 47.4 | 18.7 | 24.5 | 31.6 | 39.5 | 47.3 | 54.2 | 59.9 | 64.1 | 66.8 |
| Guinea Bissau | 2 805 | 44.3 | 17.9 | 23.1 | 29.6 | 36.8 | 44.1 | 50.6 | 55.9 | 59.8 | 62.3 |
| Guyana | 26 215 | 48.6 | 16.6 | 22.0 | 29.1 | 37.4 | 46.1 | 54.4 | 61.7 | 68.0 | 73.1 |
| Haiti | 32 371 | 49.8 | 22.6 | 27.7 | 33.9 | 41.0 | 48.2 | 55.0 | 60.9 | 65.8 | 69.9 |
| Honduras | 183 870 | 42.4 | 12.2 | 16.6 | 22.7 | 30.2 | 38.6 | 47.0 | 54.8 | 61.7 | 67.7 |
| Hungary | 4 222 | 57.2 | 21.6 | 28.5 | 37.2 | 47.0 | 56.8 | 65.6 | 72.7 | 77.9 | 81.4 |
| Iceland | 3 843 118 | 38.3 | 4.6 | 7.8 | 13.1 | 20.8 | 30.6 | 41.6 | 52.5 | 62.6 | 71.3 |
| India | 987 673 | 37.0 | 15.2 | 19.0 | 23.8 | 29.3 | 35.0 | 40.5 | 45.4 | 49.8 | 54.1 |
| Indonesia | 524 457 | 47.2 | 18.9 | 24.8 | 31.8 | 39.3 | 46.7 | 53.3 | 58.7 | 63.1 | 66.4 |
| Iran | 159 548 | 34.8 | 5.9 | 9.1 | 14.2 | 21.5 | 30.4 | 39.7 | 48.3 | 55.4 | 60.5 |
| Iraq | 53 932 | 56.3 | 22.7 | 29.4 | 37.8 | 47.2 | 56.4 | 64.4 | 70.8 | 75.4 | 78.5 |
| Ireland | 81 923 | 40.7 | 12.1 | 16.1 | 21.6 | 28.6 | 36.7 | 45.0 | 52.7 | 59.6 | 65.3 |
| Israel | 85 869 | 38.9 | 6.5 | 10.3 | 16.1 | 24.0 | 33.4 | 43.3 | 52.8 | 61.2 | 68.1 |
| Italy | 1 487 368 | 44.4 | 8.2 | 13.0 | 19.9 | 28.9 | 39.4 | 50.1 | 60.0 | 68.6 | 75.6 |
| Jamaica | 17 937 | 54.6 | 21.7 | 28.0 | 35.8 | 44.5 | 53.3 | 61.4 | 68.2 | 73.8 | 78.2 |
| Japan | 4 117 308 | 40.5 | 8.6 | 13.2 | 19.6 | 27.5 | 36.5 | 45.6 | 53.9 | 61.2 | 67.2 |
| Jordan | 37 373 | 46.6 | 12.9 | 18.2 | 25.6 | 34.8 | 44.7 | 54.1 | 62.0 | 68.0 | 72.0 |
| Kazakhstan | 107 698 | 50.8 | 15.8 | 21.8 | 29.9 | 39.5 | 49.4 | 58.6 | 66.2 | 72.0 | 76.1 |
| Kenya | 86 815 | 39.1 | 16.5 | 20.2 | 25.2 | 31.2 | 37.6 | 43.6 | 48.8 | 53.1 | 56.4 |
| Kiribati | 255 | 47.2 | 27.6 | 31.0 | 35.6 | 40.9 | 46.4 | 51.5 | 55.7 | 59.1 | 61.5 |
| Kuwait | 18 001 | 48.0 | 16.0 | 20.9 | 27.9 | 36.5 | 45.9 | 54.8 | 62.4 | 68.3 | 72.5 |
| Kyrgyzstan | 28 171 | 49.6 | 15.0 | 21.0 | 29.1 | 38.5 | 48.3 | 57.3 | 64.8 | 70.6 | 74.7 |
| Lao PDR | 19 370 | 34.4 | 11.8 | 15.9 | 21.1 | 27.1 | 33.3 | 39.1 | 44.0 | 48.0 | 50.9 |
| Latvia | 38 907 | 52.6 | 17.5 | 24.1 | 32.6 | 42.3 | 52.3 | 61.3 | 68.4 | 73.5 | 76.4 |
| Lebanon | 45 952 | 46.0 | 16.4 | 21.1 | 27.7 | 35.7 | 44.4 | 52.5 | 59.4 | 64.7 | 68.3 |
| Lesotho | 5 312 | 46.6 | 18.5 | 23.8 | 30.5 | 37.9 | 45.5 | 52.4 | 58.3 | 63.1 | 66.9 |
| Liberia | 7 084 | 46.4 | 18.0 | 23.7 | 30.6 | 38.3 | 46.0 | 53.0 | 58.6 | 62.9 | 65.6 |
| Libya | 29 286 | 52.2 | 15.8 | 21.8 | 30.0 | 40.1 | 50.7 | 60.5 | 68.5 | 74.6 | 78.9 |
| Lithuania | 56 244 | 56.5 | 20.9 | 28.2 | 37.2 | 47.3 | 57.2 | 65.8 | 72.4 | 76.9 | 79.3 |
| Luxembourg | 6 583 | 40.4 | 7.6 | 11.5 | 17.3 | 25.1 | 34.4 | 44.3 | 54.0 | 62.8 | 70.5 |
| Macedonia (TFYR) | 28 279 | 54.1 | 18.1 | 24.8 | 33.4 | 43.3 | 53.4 | 62.5 | 70.0 | 75.5 | 79.1 |
| Madagascar | 37 380 | 42.7 | 20.4 | 24.2 | 29.3 | 35.3 | 41.5 | 47.3 | 52.2 | 56.3 | 59.3 |
| Malawi | 36 636 | 37.2 | 10.0 | 13.6 | 18.9 | 25.6 | 33.2 | 41.0 | 48.3 | 54.9 | 60.9 |
| Malaysia | 142 172 | 48.9 | 16.3 | 22.8 | 30.7 | 39.5 | 48.3 | 56.1 | 62.5 | 67.6 | 71.4 |
| Maldives | 1 703 | 42.5 | 10.7 | 16.1 | 23.1 | 31.5 | 40.3 | 48.7 | 56.0 | 62.0 | 66.7 |
| Mali | 31 455 | 41.2 | 14.4 | 19.4 | 25.7 | 32.9 | 40.3 | 47.2 | 52.9 | 57.3 | 60.3 |
| Malta | 6 651 | 39.6 | 6.2 | 10.1 | 16.0 | 24.0 | 33.7 | 43.9 | 53.7 | 62.5 | 69.8 |
| Mauritania | 8 404 | 44.3 | 18.0 | 23.2 | 29.6 | 36.8 | 44.1 | 50.6 | 55.9 | 59.8 | 62.3 |
| Mauritius | 9 833 | 42.7 | 9.5 | 14.0 | 20.5 | 28.9 | 38.3 | 47.9 | 56.6 | 64.3 | 70.7 |
| Mexico | 596 202 | 39.9 | 12.6 | 16.7 | 22.2 | 28.9 | 36.4 | 44.0 | 50.9 | 57.1 | 62.6 |
| Micronesia (Federated States of) | 281 | 40.1 | 13.8 | 17.9 | 23.5 | 30.1 | 37.4 | 44.6 | 51.0 | 56.7 | 61.3 |
| Moldova | 46 924 | 56.1 | 22.0 | 29.2 | 38.1 | 47.7 | 57.1 | 65.1 | 71.2 | 75.3 | 77.5 |
| Mongolia | 10 986 | 50.7 | 19.3 | 25.2 | 32.7 | 41.3 | 50.1 | 58.0 | 64.5 | 69.4 | 72.6 |
| Montenegro | 8 247 | 54.2 | 18.2 | 24.9 | 33.5 | 43.4 | 53.5 | 62.6 | 70.0 | 75.5 | 79.2 |
| Morocco | 225 682 | 43.5 | 13.2 | 17.9 | 24.4 | 32.5 | 41.3 | 49.7 | 57.0 | 62.7 | 66.8 |
| Mozambique | 43 083 | 43.8 | 21.9 | 25.8 | 30.9 | 36.8 | 42.8 | 48.4 | 53.1 | 56.9 | 59.7 |
| Myanmar | 228 825 | 43.4 | 19.3 | 24.4 | 30.5 | 37.1 | 43.5 | 49.1 | 53.6 | 57.0 | 59.3 |
| Namibia | 7 738 | 49.7 | 25.6 | 30.5 | 36.4 | 42.8 | 49.3 | 55.0 | 59.7 | 63.4 | 66.2 |
| Nepal | 85 635 | 41.2 | 22.5 | 26.6 | 31.4 | 36.5 | 41.4 | 45.6 | 48.9 | 51.4 | 53.3 |
| Netherlands | 277 262 | 40.7 | 7.5 | 11.3 | 17.1 | 24.9 | 34.4 | 44.6 | 54.5 | 63.5 | 71.4 |
| New Zealand | 65 616 | 39.3 | 11.2 | 14.9 | 20.1 | 27.0 | 35.0 | 43.3 | 51.2 | 58.2 | 64.0 |
| Nicaragua | 28 540 | 43.6 | 14.9 | 19.6 | 25.7 | 33.0 | 40.9 | 48.5 | 55.3 | 61.3 | 66.3 |
| Niger | 26 476 | 47.3 | 22.7 | 28.0 | 34.3 | 41.1 | 47.8 | 53.6 | 58.1 | 61.3 | 63.1 |
| Nigeria | 300 332 | 42.5 | 15.6 | 21.1 | 27.9 | 35.4 | 42.8 | 49.3 | 54.5 | 58.2 | 60.0 |
| North Korea | 192 152 | 34.1 | 7.8 | 11.7 | 17.1 | 23.6 | 31.0 | 38.3 | 45.1 | 50.9 | 55.8 |
| Norway | 75 301 | 40.3 | 7.8 | 11.7 | 17.4 | 25.1 | 34.3 | 44.3 | 53.9 | 62.6 | 70.0 |
| Occupied Palestinian Territory | 2 962 640 | 48.3 | 13.6 | 19.1 | 26.7 | 36.1 | 46.3 | 55.8 | 63.9 | 70.1 | 74.5 |
| Oman | 11 965 | 51.2 | 21.0 | 26.4 | 33.5 | 41.9 | 50.5 | 58.3 | 64.6 | 69.3 | 72.4 |
| Pakistan | 374 060 | 47.7 | 28.8 | 33.0 | 38.0 | 43.1 | 47.9 | 52.0 | 55.3 | 57.8 | 59.7 |
| Panama | 26 527 | 44.2 | 15.1 | 19.6 | 25.7 | 33.1 | 41.1 | 49.0 | 56.1 | 62.3 | 67.6 |
| Papua New Guinea | 13 460 | 33.5 | 12.7 | 15.9 | 20.3 | 25.6 | 31.5 | 37.2 | 42.3 | 46.6 | 50.2 |
| Paraguay | 37 803 | 63.8 | 32.9 | 39.4 | 47.2 | 55.5 | 63.6 | 70.6 | 76.3 | 80.9 | 84.5 |
| Peru | 196 699 | 27.5 | 6.7 | 8.9 | 12.2 | 16.6 | 22.1 | 28.2 | 34.5 | 41.2 | 48.4 |
| Philippines | 328 852 | 39.4 | 16.1 | 20.9 | 26.6 | 32.9 | 39.3 | 44.9 | 49.5 | 52.9 | 55.1 |
| Poland | 663 408 | 57.8 | 22.2 | 29.1 | 37.9 | 47.8 | 57.7 | 66.5 | 73.5 | 78.6 | 81.8 |
| Portugal | 200 994 | 42.9 | 7.4 | 12.0 | 18.6 | 27.4 | 37.8 | 48.4 | 58.2 | 66.7 | 73.6 |
| Puerto Rico | 51 313 | 50.6 | 19.8 | 25.3 | 32.3 | 40.4 | 48.7 | 56.6 | 63.4 | 69.1 | 73.7 |
| Qatar | 4 201 | 48.8 | 14.4 | 19.8 | 27.3 | 36.7 | 46.8 | 56.3 | 64.3 | 70.5 | 74.8 |
| Romania | 341 195 | 56.9 | 20.9 | 28.3 | 37.4 | 47.4 | 57.3 | 65.8 | 72.6 | 77.4 | 80.4 |
| Russian Federation | 1 949 811 | 52.8 | 17.2 | 24.2 | 33.1 | 43.1 | 53.2 | 62.1 | 69.0 | 73.7 | 76.3 |
| Rwanda | 24 705 | 35.5 | 14.6 | 17.8 | 22.3 | 27.7 | 33.6 | 39.3 | 44.4 | 48.7 | 52.0 |
| Saint Lucia | 1 217 | 48.0 | 17.6 | 22.7 | 29.4 | 37.4 | 45.8 | 53.7 | 60.6 | 66.5 | 71.2 |
| Saint Vincent and the Grenadines | 830 | 48.4 | 14.9 | 20.5 | 27.8 | 36.5 | 45.7 | 54.6 | 62.4 | 69.0 | 74.4 |
| Samoa | 191 | 45.1 | 19.6 | 23.9 | 29.5 | 36.2 | 43.2 | 49.9 | 55.8 | 60.7 | 64.7 |
| Sao Tome and Principe | 418 | 51.6 | 23.4 | 29.7 | 36.9 | 44.7 | 52.1 | 58.5 | 63.6 | 67.2 | 69.5 |
| Saudi Arabia | 85 735 | 41.4 | 11.7 | 16.1 | 22.3 | 30.1 | 38.8 | 47.4 | 54.8 | 60.6 | 64.7 |
| Senegal | 29 539 | 46.9 | 19.3 | 24.9 | 31.7 | 39.2 | 46.6 | 53.3 | 58.7 | 62.8 | 65.7 |
| Serbia | 129 117 | 55.5 | 17.5 | 24.5 | 33.7 | 44.2 | 55.0 | 64.7 | 72.3 | 77.9 | 81.5 |
| Seychelles | 607 | 52.5 | 19.8 | 25.7 | 33.3 | 42.1 | 51.1 | 59.4 | 66.3 | 71.9 | 76.3 |
| Sierra Leone | 12 993 | 46.7 | 21.4 | 26.9 | 33.3 | 40.3 | 47.1 | 53.1 | 57.7 | 61.0 | 62.8 |
| Singapore | 48 906 | 41.9 | 7.1 | 11.3 | 17.4 | 25.7 | 35.6 | 46.0 | 56.1 | 65.4 | 73.6 |
| Slovakia | 77 185 | 52.6 | 14.1 | 20.9 | 29.8 | 40.3 | 51.3 | 61.4 | 69.6 | 75.9 | 80.2 |
| Slovenia | 43 038 | 54.2 | 18.3 | 24.9 | 33.5 | 43.4 | 53.5 | 62.6 | 70.0 | 75.4 | 79.1 |
| Solomon Islands | 1 276 | 37.1 | 10.8 | 14.8 | 20.2 | 26.9 | 34.3 | 41.5 | 48.1 | 53.7 | 58.4 |
| Somalia | 21 758 | 42.4 | 18.4 | 22.4 | 27.9 | 34.2 | 40.9 | 47.2 | 52.7 | 57.2 | 60.6 |
| South Africa | 241 937 | 51.3 | 22.8 | 28.3 | 35.1 | 42.7 | 50.3 | 57.2 | 63.0 | 67.8 | 71.7 |
| South Korea | 671 288 | 35.7 | 6.9 | 10.0 | 14.7 | 21.2 | 29.3 | 38.3 | 47.3 | 55.8 | 63.5 |
| South Sudan | 14 107 | 40.6 | 16.8 | 20.7 | 25.9 | 32.1 | 38.8 | 45.1 | 50.7 | 55.3 | 59.0 |
| Spain | 826 686 | 37.3 | 5.0 | 8.3 | 13.6 | 21.0 | 30.4 | 40.8 | 51.1 | 60.5 | 68.3 |
| Sri Lanka | 146 778 | 42.3 | 16.3 | 21.1 | 27.1 | 33.8 | 40.7 | 47.1 | 52.6 | 57.4 | 61.6 |
| Sudan | 115 705 | 46.3 | 24.6 | 28.6 | 33.8 | 39.6 | 45.6 | 51.0 | 55.6 | 59.1 | 61.7 |
| Suriname | 3 070 | 51.5 | 18.8 | 24.7 | 32.1 | 40.6 | 49.5 | 57.7 | 64.9 | 70.9 | 75.8 |
| Swaziland | 2 416 | 49.6 | 21.1 | 26.6 | 33.5 | 41.1 | 48.7 | 55.5 | 61.3 | 66.0 | 69.7 |
| Sweden | 153 805 | 39.9 | 7.6 | 11.6 | 17.3 | 25.0 | 34.2 | 44.0 | 53.4 | 61.9 | 69.0 |
| Switzerland | 142 105 | 31.5 | 3.2 | 5.5 | 9.5 | 15.5 | 23.4 | 32.7 | 42.5 | 52.2 | 61.2 |
| Syrian Arab Republic | 85 971 | 50.0 | 15.8 | 21.6 | 29.3 | 38.7 | 48.5 | 57.6 | 65.2 | 71.0 | 75.0 |
| Taiwan | 279 296 | 32.1 | 5.7 | 8.9 | 13.6 | 19.9 | 27.2 | 35.0 | 42.7 | 49.7 | 56.2 |
| Tajikistan | 29 261 | 55.5 | 21.0 | 27.6 | 36.0 | 45.5 | 55.1 | 63.6 | 70.5 | 75.6 | 79.0 |
| Tanzania | 110 250 | 39.1 | 16.0 | 20.0 | 25.3 | 31.5 | 38.1 | 44.2 | 49.5 | 53.4 | 56.0 |
| Thailand | 670 047 | 36.2 | 10.0 | 14.4 | 20.2 | 27.0 | 34.2 | 41.1 | 47.2 | 52.3 | 56.4 |
| Timor-Leste | 4 036 | 39.4 | 21.7 | 25.6 | 30.2 | 35.1 | 40.0 | 44.1 | 47.3 | 49.4 | 50.3 |
| Togo | 12 196 | 41.7 | 17.8 | 22.5 | 28.4 | 35.0 | 41.6 | 47.5 | 52.3 | 55.8 | 57.8 |
| Tonga | 447 | 50.6 | 22.4 | 27.4 | 33.9 | 41.4 | 49.1 | 56.3 | 62.4 | 67.4 | 71.3 |
| Trinidad and Tobago | 10 194 | 50.5 | 20.1 | 25.5 | 32.3 | 40.3 | 48.5 | 56.2 | 62.9 | 68.6 | 73.3 |
| Tunisia | 95 059 | 43.5 | 11.3 | 16.0 | 22.7 | 31.3 | 40.7 | 49.9 | 57.9 | 64.4 | 69.0 |
| Turkey | 803 590 | 41.8 | 8.8 | 13.5 | 20.2 | 28.9 | 38.7 | 48.2 | 56.6 | 63.3 | 68.3 |
| Turkmenistan | 22 117 | 47.6 | 14.6 | 20.2 | 27.6 | 36.5 | 45.9 | 54.7 | 62.1 | 67.9 | 71.9 |
| Uganda | 58 045 | 36.8 | 19.8 | 22.6 | 26.5 | 31.2 | 36.1 | 40.7 | 44.5 | 47.4 | 49.2 |
| Ukraine | 651 773 | 52.0 | 15.5 | 22.5 | 31.4 | 41.7 | 52.0 | 61.2 | 68.5 | 73.6 | 76.6 |
| United Arab Emirates | 11 711 | 47.0 | 17.0 | 22.0 | 28.8 | 37.0 | 45.6 | 53.7 | 60.5 | 65.6 | 69.1 |
| United Kingdom | 907 331 | 34.9 | 7.5 | 10.7 | 15.4 | 21.8 | 29.6 | 38.0 | 46.3 | 53.9 | 60.5 |
| United States of America | 5 268 893 | 40.4 | 10.6 | 14.5 | 20.0 | 27.2 | 35.7 | 44.5 | 52.9 | 60.3 | 66.6 |
| Uruguay | 47 160 | 51.9 | 16.8 | 22.7 | 30.5 | 39.6 | 49.3 | 58.5 | 66.5 | 73.2 | 78.7 |
| Uzbekistan | 108 010 | 52.7 | 23.9 | 29.5 | 36.7 | 44.7 | 52.7 | 59.8 | 65.5 | 69.6 | 72.2 |
| Vanuatu | 696 | 46.5 | 20.1 | 24.9 | 31.0 | 37.9 | 45.1 | 51.8 | 57.5 | 62.2 | 66.0 |
| Venezuela | 153 345 | 48.3 | 16.1 | 21.4 | 28.3 | 36.5 | 45.2 | 53.7 | 61.3 | 68.0 | 73.7 |
| Vietnam | 531 633 | 37.2 | 10.0 | 14.3 | 20.1 | 27.0 | 34.6 | 42.0 | 48.5 | 54.2 | 58.8 |
| Yemen | 75 189 | 35.6 | 12.1 | 15.7 | 20.9 | 27.1 | 34.0 | 40.7 | 46.5 | 50.9 | 53.7 |
| Zambia | 26 055 | 38.6 | 14.6 | 18.3 | 23.4 | 29.7 | 36.5 | 43.1 | 48.9 | 53.8 | 57.7 |
| Zimbabwe | 27 377 | 48.5 | 21.7 | 26.9 | 33.3 | 40.4 | 47.6 | 54.0 | 59.5 | 64.0 | 67.4 |

HTN: Hypertension.

**Supplementary Table 3 – Male and female population attributable fractions of hypertension for dementia (based on age at hypertension diagnosis) globally and by region (%).**

| Region | 30-44 (95% CI) | | 45-54 (95% CI) | | 55-64 (95% CI) | | 65-74 (95% CI) | | Total (95% CI) | |
| --- | --- | --- | --- | --- | --- | --- | --- | --- | --- | --- |
|  | Male | Female | Male | Female | Male | Female | Male | Female | Male | Female |
| Global | 9.72 (3.08 to 16.36) | 7.75 (3.34 to 12.15) | 2.67 (0.82 to 4.51) | 3.06 (0.98 to 5.14) | 2.25 (0.92 to 3.57) | 2.77 (1.18 to 4.36) | 1.36 (-1.78 to 4.49) | 2.07 (-2.68 to 6.82) | 15.99 (8.37 to 23.61) | 15.65 (8.72 to 22.58) |
| Africa | 9.04 (1.94 to 16.13) | 10.91 (3.86 to 17.96) | 2.35 (0.71 to 3.99) | 3.07 (0.98 to 5.17) | 2.07 (0.84 to 3.30) | 2.20 (0.90 to 3.50) | 1.25 (-1.58 to 4.07) | 1.22 (-1.54 to 3.98) | 14.70 (6.85 to 22.55) | 17.40 (9.51 to 25.30) |
| Asia | 9.48 (2.74 to 16.23) | 7.61 (3.24 to 11.98) | 2.46 (0.59 to 4.33) | 2.99 (0.85 to 5.12) | 2.02 (0.58 to 3.45) | 2.60 (0.89 to 4.30) | 1.20 (-1.82 to 4.21) | 1.86 (-2.63 to 6.35) | 15.16 (7.63 to 22.68) | 15.05 (8.45 to 21.66) |
| Europe | 10.75 (4.05 to 17.44) | 7.07 (3.04 to 11.10) | 3.39 (1.06 to 5.72) | 3.31 (1.05 to 5.58) | 2.86 (1.21 to 4.52) | 3.33 (1.44 to 5.22) | 1.68 (-2.14 to 5.50) | 2.64 (-3.21 to 8.48) | 18.68 (10.56 to 26.80) | 16.35 (8.73 to 23.97) |
| LA and Caribbean | 11.13 (2.21 to 20.06) | 10.13 (3.68 to 16.59) | 2.52 (0.56 to 4.48) | 3.33 (0.92 to 5.74) | 2.14 (0.68 to 3.60) | 2.80 (1.06 to 4.54) | 1.50 (-2.02 to 5.01) | 2.08 (-2.69 to 6.85) | 17.29 (7.55 to 27.02) | 18.34 (9.97 to 26.71) |
| Northern America | 8.61 (1.91 to 15.31) | 6.57 (1.82 to 11.31) | 2.83 (0.32 to 5.33) | 2.87 (0.46 to 5.29) | 2.65 (0.51 to 4.79) | 3.04 (0.77 to 5.32) | 1.69 (-2.75 to 6.13) | 2.70 (-3.75 to 9.15) | 15.78 (7.75 to 23.81) | 15.18 (6.98 to 23.38) |
| Oceania | 8.34 (2.11 to 14.56) | 6.29 (1.65 to 10.93) | 2.89 (0.43 to 5.36) | 2.62 (0.38 to 4.87) | 2.76 (0.63 to 4.90) | 2.80 (0.64 to 4.95) | 1.80 (-2.84 to 6.44) | 2.57 (-3.57 to 8.70) | 15.79 (7.96 to 23.63) | 14.28 (6.45 to 22.12) |

## **Supplementary Table 4 – Population Attributable Fraction of hypertension for dementia by country and gender (%).**

| Country | PAF (95% CI) | PAF Male (95% CI) | PAF Female (95% CI) |
| --- | --- | --- | --- |
| Afghanistan | 16.49 (5.70 to 27.28) | 14.52 (4.04 to 24.99) | 17.56 (5.14 to 29.97) |
| Albania | 19.29 (10.12 to 28.46) | 18.27 (7.44 to 29.10) | 19.84 (10.29 to 29.40) |
| Algeria | 17.96 (9.53 to 26.39) | 16.09 (6.55 to 25.63) | 18.96 (9.90 to 28.03) |
| Angola | 16.85 (5.79 to 27.91) | 15.77 (2.92 to 28.63) | 17.43 (4.44 to 30.42) |
| Antigua and Barbuda | 19.07 (7.10 to 31.04) | 17.51 (2.79 to 32.23) | 19.91 (6.24 to 33.57) |
| Argentina | 19.84 (9.36 to 30.31) | 20.69 (7.27 to 34.11) | 19.38 (9.27 to 29.50) |
| Armenia | 20.67 (10.12 to 31.22) | 19.58 (6.92 to 32.25) | 21.26 (10.41 to 32.11) |
| Australia | 14.80 (7.12 to 22.48) | 15.99 (7.84 to 24.13) | 14.16 (6.04 to 22.29) |
| Austria | 16.77 (7.37 to 26.17) | 18.12 (6.73 to 29.52) | 16.04 (5.39 to 26.69) |
| Azerbaijan | 19.08 (9.63 to 28.52) | 17.85 (7.65 to 28.06) | 19.73 (9.43 to 30.04) |
| Bahamas | 18.32 (6.55 to 30.10) | 18.35 (4.70 to 32.00) | 18.31 (5.22 to 31.40) |
| Bahrain | 17.56 (7.61 to 27.51) | 17.21 (4.12 to 30.29) | 17.75 (6.71 to 28.78) |
| Bangladesh | 12.52 (4.81 to 20.24) | 10.01 (2.97 to 17.05) | 13.88 (4.95 to 22.80) |
| Barbados | 20.08 (9.78 to 30.38) | 18.51 (6.87 to 30.16) | 20.93 (9.53 to 32.33) |
| Belarus | 22.48 (12.54 to 32.43) | 21.94 (9.78 to 34.11) | 22.77 (12.76 to 32.78) |
| Belgium | 15.68 (7.72 to 23.63) | 17.13 (8.57 to 25.69) | 14.89 (6.30 to 23.48) |
| Belize | 16.98 (5.93 to 28.03) | 15.59 (1.72 to 29.46) | 17.73 (5.32 to 30.14) |
| Benin | 14.96 (7.57 to 22.34) | 11.98 (3.59 to 20.38) | 16.56 (8.46 to 24.65) |
| Bhutan | 17.02 (6.40 to 27.64) | 15.25 (2.94 to 27.57) | 17.97 (7.18 to 28.77) |
| Bolivia | 14.12 (4.37 to 23.87) | 13.43 (0.21 to 26.65) | 14.49 (3.33 to 25.65) |
| Bosnia and Herzegovina | 21.03 (11.19 to 30.86) | 21.01 (8.75 to 33.26) | 21.04 (10.65 to 31.43) |
| Botswana | 18.18 (6.59 to 29.77) | 15.71 (2.98 to 28.44) | 19.50 (6.58 to 32.43) |
| Brazil | 19.60 (9.59 to 29.60) | 18.98 (6.95 to 31.01) | 19.93 (10.01 to 29.84) |
| Brunei Darussalam | 20.60 (10.28 to 30.91) | 19.21 (6.97 to 31.45) | 21.34 (10.70 to 31.99) |
| Bulgaria | 20.42 (9.35 to 31.50) | 20.79 (5.62 to 35.97) | 20.23 (8.29 to 32.16) |
| Burkina Faso | 13.70 (5.08 to 22.33) | 12.49 (2.51 to 22.47) | 14.36 (4.28 to 24.43) |
| Burundi | 15.18 (4.32 to 26.03) | 13.34 (-0.23 to 26.91) | 16.17 (3.38 to 28.95) |
| Cabo Verde | 19.55 (10.20 to 28.89) | 17.65 (5.63 to 29.66) | 20.57 (11.26 to 29.88) |
| Cambodia | 12.40 (4.48 to 20.32) | 11.74 (2.10 to 21.38) | 12.75 (3.45 to 22.05) |
| Cameroon | 17.07 (7.71 to 26.44) | 15.64 (5.19 to 26.10) | 17.84 (7.17 to 28.52) |
| Canada | 12.47 (5.20 to 19.75) | 13.97 (6.42 to 21.51) | 11.67 (4.16 to 19.18) |
| Central African Republic | 19.42 (9.71 to 29.12) | 18.21 (7.45 to 28.97) | 20.06 (9.32 to 30.81) |
| Chad | 16.41 (5.29 to 27.53) | 14.71 (1.97 to 27.44) | 17.33 (4.13 to 30.53) |
| Chile | 18.02 (9.33 to 26.70) | 18.48 (8.15 to 28.80) | 17.77 (8.79 to 26.74) |
| China | 13.56 (7.11 to 20.02) | 13.72 (5.82 to 21.62) | 13.47 (6.61 to 20.34) |
| Colombia | 15.98 (7.47 to 24.49) | 14.72 (4.71 to 24.74) | 16.66 (7.32 to 25.99) |
| Comoros | 15.19 (5.67 to 24.72) | 13.12 (2.57 to 23.68) | 16.31 (5.11 to 27.50) |
| Congo | 17.60 (6.06 to 29.14) | 16.41 (2.81 to 30.01) | 18.24 (4.55 to 31.93) |
| Costa Rica | 18.33 (8.97 to 27.69) | 16.68 (6.33 to 27.03) | 19.22 (9.09 to 29.34) |
| Cote d'Ivoire | 16.60 (5.95 to 27.24) | 16.24 (3.96 to 28.52) | 16.79 (4.34 to 29.23) |
| Croatia | 22.13 (11.75 to 32.50) | 22.37 (9.82 to 34.93) | 21.99 (11.31 to 32.68) |
| Cuba | 17.90 (7.48 to 28.32) | 16.32 (3.32 to 29.32) | 18.75 (7.33 to 30.17) |
| Cyprus | 15.97 (6.94 to 25.01) | 17.84 (6.44 to 29.25) | 14.97 (4.66 to 25.27) |
| Czech Republic | 19.59 (11.05 to 28.12) | 22.61 (11.97 to 33.25) | 17.96 (9.48 to 26.43) |
| Denmark | 16.28 (7.98 to 24.58) | 18.60 (7.74 to 29.45) | 15.03 (6.78 to 23.28) |
| Djibouti | 15.25 (4.29 to 26.21) | 13.41 (-0.34 to 27.15) | 16.24 (3.37 to 29.11) |
| Dominican Republic | 20.98 (9.03 to 32.94) | 19.61 (5.52 to 33.69) | 21.73 (8.80 to 34.65) |
| DR Congo | 15.71 (5.14 to 26.29) | 14.84 (2.51 to 27.17) | 16.18 (3.35 to 29.01) |
| Ecuador | 13.52 (6.19 to 20.84) | 13.21 (4.43 to 21.98) | 13.68 (5.91 to 21.46) |
| Egypt | 17.92 (9.17 to 26.67) | 15.55 (6.30 to 24.80) | 19.20 (9.76 to 28.64) |
| El Salvador | 15.70 (7.18 to 24.21) | 14.12 (4.29 to 23.95) | 16.55 (7.14 to 25.95) |
| Equatorial Guinea | 17.08 (4.97 to 29.20) | 15.84 (1.50 to 30.19) | 17.75 (3.14 to 32.37) |
| Eritrea | 11.48 (3.90 to 19.06) | 9.79 (0.39 to 19.19) | 12.39 (3.59 to 21.18) |
| Estonia | 17.58 (8.42 to 26.74) | 18.06 (4.94 to 31.17) | 17.33 (7.83 to 26.82) |
| Ethiopia | 11.64 (3.33 to 19.95) | 10.27 (1.38 to 19.15) | 12.37 (2.57 to 22.18) |
| Fiji | 17.89 (7.53 to 28.25) | 15.16 (2.62 to 27.70) | 19.36 (7.62 to 31.10) |
| Finland | 17.49 (9.32 to 25.67) | 18.88 (9.31 to 28.46) | 16.75 (8.07 to 25.42) |
| France | 15.77 (7.65 to 23.89) | 18.47 (9.60 to 27.34) | 14.32 (5.66 to 22.97) |
| Gabon | 16.66 (5.80 to 27.51) | 15.60 (2.77 to 28.43) | 17.23 (4.51 to 29.94) |
| Gambia | 16.88 (6.66 to 27.10) | 15.11 (3.73 to 26.48) | 17.83 (5.93 to 29.73) |
| Georgia | 19.95 (10.25 to 29.64) | 18.54 (6.13 to 30.95) | 20.71 (10.87 to 30.54) |
| Germany | 15.67 (7.38 to 23.96) | 17.66 (8.12 to 27.20) | 14.60 (5.60 to 23.60) |
| Ghana | 15.56 (7.08 to 24.03) | 13.47 (3.98 to 22.95) | 16.68 (7.17 to 26.20) |
| Greece | 16.43 (8.05 to 24.81) | 18.39 (9.29 to 27.49) | 15.38 (6.40 to 24.36) |
| Grenada | 19.91 (8.34 to 31.48) | 18.10 (3.28 to 32.92) | 20.89 (8.61 to 33.17) |
| Guatemala | 15.75 (7.11 to 24.40) | 14.11 (3.73 to 24.49) | 16.64 (6.97 to 26.31) |
| Guinea | 18.21 (7.46 to 28.97) | 15.69 (2.70 to 28.68) | 19.57 (7.46 to 31.68) |
| Guinea Bissau | 16.82 (5.51 to 28.13) | 15.11 (1.20 to 29.03) | 17.74 (4.40 to 31.08) |
| Guyana | 18.84 (9.07 to 28.60) | 17.02 (6.02 to 28.02) | 19.81 (9.20 to 30.43) |
| Haiti | 18.17 (7.27 to 29.07) | 16.32 (5.69 to 26.94) | 19.17 (6.75 to 31.59) |
| Honduras | 16.83 (6.97 to 26.68) | 15.35 (3.29 to 27.42) | 17.62 (6.42 to 28.82) |
| Hungary | 20.88 (9.28 to 32.48) | 22.06 (5.98 to 38.15) | 20.24 (8.11 to 32.37) |
| Iceland | 15.37 (6.79 to 23.95) | 17.03 (7.85 to 26.21) | 14.48 (5.08 to 23.88) |
| India | 14.02 (6.34 to 21.70) | 12.51 (3.41 to 21.61) | 14.83 (6.89 to 22.77) |
| Indonesia | 18.19 (8.64 to 27.75) | 15.66 (6.35 to 24.97) | 19.56 (8.99 to 30.13) |
| Iran | 14.30 (7.80 to 20.81) | 13.25 (6.79 to 19.71) | 14.87 (7.87 to 21.88) |
| Iraq | 20.76 (9.78 to 31.74) | 20.14 (7.54 to 32.75) | 21.09 (9.53 to 32.65) |
| Ireland | 15.24 (6.43 to 24.05) | 17.10 (5.76 to 28.44) | 14.24 (4.53 to 23.95) |
| Israel | 15.53 (7.50 to 23.55) | 16.90 (8.19 to 25.62) | 14.79 (6.00 to 23.58) |
| Italy | 17.55 (9.26 to 25.85) | 19.14 (10.11 to 28.18) | 16.70 (7.76 to 25.64) |
| Jamaica | 20.55 (9.02 to 32.09) | 18.65 (5.42 to 31.87) | 21.58 (8.84 to 34.33) |
| Japan | 15.42 (8.16 to 22.68) | 19.26 (10.61 to 27.92) | 13.34 (5.80 to 20.89) |
| Jordan | 18.30 (10.33 to 26.27) | 17.39 (7.90 to 26.88) | 18.79 (10.62 to 26.97) |
| Kazakhstan | 19.80 (10.63 to 28.97) | 18.47 (8.27 to 28.67) | 20.51 (10.89 to 30.14) |
| Kenya | 14.76 (6.06 to 23.46) | 12.34 (2.03 to 22.65) | 16.06 (6.54 to 25.57) |
| Kiribati | 15.68 (3.72 to 27.64) | 14.30 (1.10 to 27.50) | 16.42 (3.13 to 29.71) |
| Kuwait | 18.12 (9.40 to 26.84) | 17.82 (6.26 to 29.38) | 18.28 (9.45 to 27.12) |
| Kyrgyzstan | 19.58 (9.97 to 29.18) | 17.85 (7.56 to 28.14) | 20.50 (9.80 to 31.21) |
| Lao PDR | 13.51 (5.06 to 21.97) | 12.45 (3.70 to 21.21) | 14.08 (3.74 to 24.42) |
| Latvia | 19.89 (9.88 to 29.90) | 20.15 (6.36 to 33.94) | 19.75 (9.32 to 30.18) |
| Lebanon | 17.22 (8.68 to 25.75) | 17.04 (5.67 to 28.42) | 17.31 (8.80 to 25.83) |
| Lesotho | 18.29 (8.15 to 28.44) | 13.77 (3.46 to 24.09) | 20.73 (9.01 to 32.45) |
| Liberia | 17.44 (6.99 to 27.89) | 16.97 (5.84 to 28.10) | 17.69 (5.45 to 29.93) |
| Libya | 20.07 (10.39 to 29.76) | 19.65 (6.78 to 32.52) | 20.30 (10.11 to 30.50) |
| Lithuania | 20.98 (10.38 to 31.59) | 21.22 (6.37 to 36.06) | 20.86 (10.04 to 31.68) |
| Luxembourg | 15.66 (7.49 to 23.82) | 18.41 (9.25 to 27.58) | 14.17 (5.48 to 22.87) |
| Macedonia (TFYR) | 20.42 (9.43 to 31.41) | 20.79 (5.76 to 35.83) | 20.22 (8.49 to 31.95) |
| Madagascar | 15.40 (4.03 to 26.77) | 13.63 (0.07 to 27.20) | 16.35 (3.15 to 29.56) |
| Malawi | 15.20 (7.49 to 22.92) | 12.54 (4.00 to 21.08) | 16.64 (8.21 to 25.07) |
| Malaysia | 19.13 (9.87 to 28.39) | 17.85 (7.27 to 28.44) | 19.82 (9.89 to 29.75) |
| Maldives | 17.39 (8.25 to 26.54) | 15.58 (4.67 to 26.49) | 18.37 (7.91 to 28.84) |
| Mali | 16.13 (6.02 to 26.25) | 14.46 (3.61 to 25.32) | 17.03 (4.81 to 29.25) |
| Malta | 15.70 (7.44 to 23.96) | 17.61 (8.41 to 26.82) | 14.67 (5.58 to 23.76) |
| Mauritania | 16.81 (5.38 to 28.24) | 15.11 (1.67 to 28.54) | 17.72 (4.11 to 31.33) |
| Mauritius | 17.07 (8.94 to 25.20) | 16.94 (7.46 to 26.41) | 17.15 (8.70 to 25.60) |
| Mexico | 15.62 (7.96 to 23.28) | 14.19 (5.49 to 22.89) | 16.39 (8.43 to 24.35) |
| Micronesia (Federated States of) | 15.47 (6.70 to 24.24) | 14.43 (4.41 to 24.46) | 16.03 (6.26 to 25.81) |
| Moldova | 20.99 (10.19 to 31.80) | 19.90 (6.57 to 33.24) | 21.58 (10.29 to 32.86) |
| Mongolia | 18.88 (9.56 to 28.21) | 18.77 (7.90 to 29.63) | 18.95 (9.49 to 28.40) |
| Montenegro | 20.44 (9.29 to 31.59) | 20.85 (5.41 to 36.29) | 20.21 (8.29 to 32.14) |
| Morocco | 17.01 (8.66 to 25.35) | 15.73 (6.06 to 25.40) | 17.69 (8.76 to 26.63) |
| Mozambique | 15.75 (5.29 to 26.21) | 13.16 (1.66 to 24.66) | 17.14 (5.26 to 29.03) |
| Myanmar | 16.22 (6.47 to 25.97) | 14.49 (4.18 to 24.80) | 17.16 (6.10 to 28.21) |
| Namibia | 17.30 (5.38 to 29.23) | 16.40 (3.08 to 29.73) | 17.78 (4.50 to 31.07) |
| Nepal | 14.26 (5.46 to 23.06) | 13.50 (2.51 to 24.50) | 14.67 (6.21 to 23.13) |
| Netherlands | 15.81 (7.50 to 24.12) | 18.39 (8.92 to 27.86) | 14.41 (5.59 to 23.24) |
| New Zealand | 15.02 (7.16 to 22.88) | 15.84 (7.12 to 24.56) | 14.57 (6.17 to 22.97) |
| Nicaragua | 16.88 (6.37 to 27.39) | 15.43 (2.63 to 28.23) | 17.66 (5.52 to 29.79) |
| Niger | 17.06 (5.28 to 28.84) | 15.77 (2.31 to 29.23) | 17.75 (4.18 to 31.32) |
| Nigeria | 16.61 (8.19 to 25.03) | 14.68 (6.15 to 23.22) | 17.64 (8.29 to 27.00) |
| North Korea | 13.53 (5.43 to 21.63) | 14.55 (4.83 to 24.27) | 12.98 (3.44 to 22.52) |
| Norway | 15.69 (7.11 to 24.28) | 17.85 (7.70 to 27.99) | 14.53 (5.16 to 23.90) |
| Occupied Palestinian Territory | 18.84 (9.55 to 28.13) | 18.34 (6.93 to 29.74) | 19.11 (9.10 to 29.13) |
| Oman | 18.61 (9.17 to 28.05) | 18.30 (5.53 to 31.07) | 18.77 (9.64 to 27.91) |
| Pakistan | 16.11 (3.96 to 28.26) | 13.57 (-0.68 to 27.83) | 17.48 (4.14 to 30.82) |
| Panama | 17.04 (7.25 to 26.82) | 15.60 (2.97 to 28.22) | 17.81 (7.18 to 28.44) |
| Papua New Guinea | 12.95 (3.33 to 22.56) | 11.17 (-0.21 to 22.55) | 13.91 (2.11 to 25.71) |
| Paraguay | 21.87 (8.62 to 35.13) | 21.19 (4.04 to 38.34) | 22.24 (8.80 to 35.68) |
| Peru | 10.88 (4.61 to 17.15) | 10.96 (4.00 to 17.91) | 10.84 (4.45 to 17.24) |
| Philippines | 14.89 (6.65 to 23.13) | 13.89 (3.69 to 24.09) | 15.43 (6.72 to 24.14) |
| Poland | 21.26 (11.33 to 31.18) | 21.35 (8.07 to 34.62) | 21.21 (11.73 to 30.68) |
| Portugal | 17.04 (8.62 to 25.46) | 18.59 (9.19 to 27.99) | 16.20 (7.00 to 25.40) |
| Puerto Rico | 19.05 (7.02 to 31.08) | 17.48 (2.60 to 32.37) | 19.89 (6.15 to 33.64) |
| Qatar | 19.00 (9.79 to 28.22) | 17.89 (6.15 to 29.64) | 19.60 (9.83 to 29.36) |
| Romania | 21.21 (11.14 to 31.28) | 21.63 (9.09 to 34.17) | 20.98 (10.94 to 31.02) |
| Russian Federation | 20.30 (11.22 to 29.37) | 19.91 (8.59 to 31.23) | 20.50 (11.39 to 29.62) |
| Rwanda | 13.39 (4.53 to 22.25) | 11.43 (0.50 to 22.37) | 14.44 (4.36 to 24.52) |
| Saint Lucia | 18.50 (8.22 to 28.78) | 15.82 (3.04 to 28.59) | 19.95 (8.77 to 31.13) |
| Saint Vincent and the Grenadines | 19.15 (9.41 to 28.90) | 17.00 (5.89 to 28.11) | 20.31 (9.65 to 30.96) |
| Samoa | 16.38 (6.01 to 26.75) | 15.76 (3.95 to 27.57) | 16.71 (5.15 to 28.26) |
| Sao Tome and Principe | 19.02 (8.44 to 29.60) | 17.07 (6.05 to 28.09) | 20.07 (8.57 to 31.58) |
| Saudi Arabia | 15.99 (7.85 to 24.13) | 16.19 (6.15 to 26.24) | 15.88 (7.15 to 24.61) |
| Senegal | 17.78 (6.97 to 28.60) | 15.69 (3.42 to 27.95) | 18.91 (6.49 to 31.33) |
| Serbia | 21.25 (11.58 to 30.92) | 20.86 (8.26 to 33.45) | 21.46 (11.62 to 31.30) |
| Seychelles | 19.82 (9.65 to 30.00) | 18.17 (5.02 to 31.33) | 20.71 (10.23 to 31.18) |
| Sierra Leone | 17.18 (5.94 to 28.43) | 15.43 (3.13 to 27.74) | 18.12 (4.91 to 31.34) |
| Singapore | 16.69 (8.30 to 25.07) | 17.96 (8.86 to 27.07) | 16.00 (7.16 to 24.85) |
| Slovakia | 20.29 (10.80 to 29.78) | 21.57 (9.77 to 33.37) | 19.60 (9.52 to 29.69) |
| Slovenia | 20.40 (9.23 to 31.57) | 20.83 (5.84 to 35.82) | 20.17 (8.03 to 32.31) |
| Solomon Islands | 15.29 (6.85 to 23.74) | 11.73 (2.96 to 20.49) | 17.21 (7.20 to 27.22) |
| Somalia | 15.88 (5.50 to 26.25) | 13.38 (0.66 to 26.10) | 17.22 (5.53 to 28.91) |
| South Africa | 18.81 (8.84 to 28.78) | 17.19 (5.85 to 28.53) | 19.68 (9.59 to 29.77) |
| South Korea | 14.00 (6.44 to 21.57) | 15.42 (7.45 to 23.38) | 13.24 (4.96 to 21.53) |
| South Sudan | 15.26 (4.41 to 26.11) | 13.31 (-0.10 to 26.71) | 16.31 (3.54 to 29.08) |
| Spain | 14.67 (6.63 to 22.70) | 17.38 (9.09 to 25.66) | 13.21 (4.14 to 22.28) |
| Sri Lanka | 16.39 (7.15 to 25.63) | 14.05 (3.23 to 24.87) | 17.65 (7.44 to 27.87) |
| Sudan | 16.11 (5.20 to 27.03) | 14.20 (2.09 to 26.32) | 17.14 (5.16 to 29.12) |
| Suriname | 19.56 (8.63 to 30.48) | 18.28 (5.39 to 31.17) | 20.24 (8.24 to 32.25) |
| Swaziland | 18.88 (8.12 to 29.64) | 15.14 (3.22 to 27.06) | 20.89 (8.87 to 32.92) |
| Sweden | 15.53 (7.51 to 23.55) | 17.78 (8.67 to 26.88) | 14.32 (5.73 to 22.91) |
| Switzerland | 12.41 (4.64 to 20.18) | 15.32 (6.64 to 24.01) | 10.84 (2.74 to 18.95) |
| Syrian Arab Republic | 19.27 (9.14 to 29.40) | 18.39 (5.60 to 31.19) | 19.74 (8.62 to 30.87) |
| Taiwan | 12.94 (5.71 to 20.17) | 13.84 (5.33 to 22.35) | 12.45 (4.44 to 20.47) |
| Tajikistan | 20.57 (10.69 to 30.45) | 20.46 (7.36 to 33.55) | 20.63 (11.07 to 30.18) |
| Tanzania | 14.74 (6.04 to 23.44) | 13.10 (3.59 to 22.60) | 15.63 (5.75 to 25.50) |
| Thailand | 14.60 (6.53 to 22.66) | 13.68 (4.01 to 23.35) | 15.09 (5.82 to 24.36) |
| Timor-Leste | 13.50 (2.89 to 24.12) | 13.06 (1.69 to 24.44) | 13.74 (1.41 to 26.08) |
| Togo | 15.67 (5.54 to 25.81) | 13.88 (1.87 to 25.88) | 16.64 (4.90 to 28.37) |
| Tonga | 18.84 (8.33 to 29.35) | 15.51 (4.02 to 27.00) | 20.63 (9.30 to 31.96) |
| Trinidad and Tobago | 18.74 (7.79 to 29.68) | 17.93 (4.73 to 31.13) | 19.17 (7.15 to 31.19) |
| Tunisia | 17.40 (9.00 to 25.80) | 15.80 (5.77 to 25.84) | 18.26 (9.18 to 27.34) |
| Turkey | 17.18 (9.45 to 24.91) | 15.22 (6.95 to 23.49) | 18.24 (9.92 to 26.56) |
| Turkmenistan | 18.56 (9.74 to 27.37) | 17.48 (8.05 to 26.91) | 19.14 (9.68 to 28.59) |
| Uganda | 12.80 (3.11 to 22.49) | 11.08 (-0.19 to 22.35) | 13.72 (2.87 to 24.58) |
| Ukraine | 20.24 (11.30 to 29.17) | 20.14 (9.76 to 30.52) | 20.29 (11.10 to 29.48) |
| United Arab Emirates | 17.40 (8.73 to 26.07) | 17.97 (6.68 to 29.25) | 17.10 (8.49 to 25.71) |
| United Kingdom | 13.70 (6.69 to 20.72) | 14.86 (7.73 to 21.99) | 13.08 (5.66 to 20.50) |
| United States of America | 15.72 (7.86 to 23.57) | 15.98 (7.65 to 24.31) | 15.57 (7.17 to 23.98) |
| Uruguay | 19.76 (9.95 to 29.57) | 19.79 (7.48 to 32.11) | 19.74 (9.62 to 29.86) |
| Uzbekistan | 18.99 (8.68 to 29.30) | 18.06 (6.03 to 30.10) | 19.49 (9.06 to 29.93) |
| Vanuatu | 17.32 (6.17 to 28.46) | 15.19 (2.45 to 27.93) | 18.47 (5.72 to 31.21) |
| Venezuela | 18.63 (9.15 to 28.10) | 17.42 (6.41 to 28.44) | 19.27 (9.30 to 29.25) |
| Vietnam | 14.66 (7.25 to 22.07) | 15.06 (5.82 to 24.31) | 14.44 (6.44 to 22.45) |
| Yemen | 13.59 (4.95 to 22.22) | 13.42 (3.46 to 23.38) | 13.68 (3.38 to 23.97) |
| Zambia | 14.99 (6.72 to 23.25) | 12.14 (2.30 to 21.97) | 16.52 (7.48 to 25.57) |
| Zimbabwe | 18.06 (5.48 to 30.65) | 15.16 (0.94 to 29.38) | 19.63 (4.64 to 34.61) |

## **Supplementary Table 5 – Population Attributable Fraction of hypertension for dementia based on age at diagnosis by country.**

| Country | 30-44 (95% CI) | 45-54 (95% CI) | 55-64 (95% CI) | 65-74 (95% CI) | Total (95% CI) |
| --- | --- | --- | --- | --- | --- |
| Afghanistan | 11.85 (1.41 to 22.29) | 2.18 (-0.34 to 4.70) | 1.48 (-0.59 to 3.55) | 0.98 (-2.79 to 4.74) | 16.49 (5.70 to 27.28) |
| Albania | 11.67 (3.52 to 19.83) | 3.55 (0.58 to 6.52) | 2.67 (0.34 to 5.00) | 1.40 (-2.73 to 5.54) | 19.29 (10.12 to 28.46) |
| Algeria | 9.91 (2.92 to 16.89) | 3.51 (0.59 to 6.43) | 2.86 (0.50 to 5.23) | 1.69 (-2.87 to 6.24) | 17.96 (9.53 to 26.39) |
| Angola | 11.28 (0.59 to 21.98) | 2.63 (-0.84 to 6.11) | 1.87 (-1.24 to 4.99) | 1.06 (-4.01 to 6.13) | 16.85 (5.79 to 27.91) |
| Antigua and Barbuda | 11.77 (0.01 to 23.54) | 3.16 (-0.75 to 7.07) | 2.49 (-0.88 to 5.85) | 1.66 (-3.77 to 7.08) | 19.07 (7.10 to 31.04) |
| Argentina | 12.61 (3.03 to 22.18) | 3.15 (0.41 to 5.89) | 2.44 (0.38 to 4.49) | 1.64 (-2.59 to 5.88) | 19.84 (9.36 to 30.31) |
| Armenia | 13.29 (3.35 to 23.24) | 3.55 (0.42 to 6.68) | 2.52 (0.24 to 4.80) | 1.31 (-2.56 to 5.18) | 20.67 (10.12 to 31.22) |
| Australia | 6.85 (1.99 to 11.71) | 2.74 (0.49 to 4.98) | 2.84 (0.78 to 4.90) | 2.37 (-3.32 to 8.06) | 14.80 (7.12 to 22.48) |
| Austria | 8.19 (0.53 to 15.85) | 3.17 (-0.15 to 6.48) | 3.08 (-0.12 to 6.27) | 2.33 (-3.95 to 8.62) | 16.77 (7.37 to 26.17) |
| Azerbaijan | 11.46 (3.02 to 19.90) | 3.45 (0.47 to 6.43) | 2.66 (0.37 to 4.95) | 1.51 (-2.78 to 5.81) | 19.08 (9.63 to 28.52) |
| Bahamas | 12.11 (0.70 to 23.53) | 2.74 (-0.54 to 6.02) | 2.10 (-0.68 to 4.87) | 1.37 (-3.33 to 6.08) | 18.32 (6.55 to 30.10) |
| Bahrain | 10.04 (0.84 to 19.24) | 3.31 (-0.28 to 6.90) | 2.67 (-0.44 to 5.78) | 1.54 (-3.64 to 6.73) | 17.56 (7.61 to 27.51) |
| Bangladesh | 8.80 (1.47 to 16.12) | 1.75 (-0.03 to 3.52) | 1.20 (-0.29 to 2.69) | 0.78 (-2.06 to 3.63) | 12.52 (4.81 to 20.24) |
| Barbados | 11.69 (2.29 to 21.10) | 3.60 (0.07 to 7.14) | 2.87 (0.04 to 5.71) | 1.91 (-3.29 to 7.12) | 20.08 (9.78 to 30.38) |
| Belarus | 14.06 (4.86 to 23.26) | 4.26 (0.88 to 7.65) | 2.87 (0.59 to 5.15) | 1.29 (-2.32 to 4.90) | 22.48 (12.54 to 32.43) |
| Belgium | 6.85 (2.16 to 11.55) | 3.05 (0.59 to 5.51) | 3.19 (0.91 to 5.46) | 2.59 (-3.53 to 8.70) | 15.68 (7.72 to 23.63) |
| Belize | 10.34 (-0.18 to 20.85) | 2.78 (-0.75 to 6.31) | 2.28 (-0.90 to 5.45) | 1.59 (-3.79 to 6.97) | 16.98 (5.93 to 28.03) |
| Benin | 8.85 (2.54 to 15.16) | 2.75 (0.38 to 5.11) | 2.15 (0.15 to 4.16) | 1.21 (-2.57 to 4.99) | 14.96 (7.57 to 22.34) |
| Bhutan | 12.26 (2.01 to 22.50) | 2.25 (0.03 to 4.46) | 1.51 (-0.23 to 3.25) | 1.01 (-2.29 to 4.30) | 17.02 (6.40 to 27.64) |
| Bolivia | 7.08 (-1.09 to 15.25) | 2.50 (-0.95 to 5.95) | 2.44 (-1.10 to 5.98) | 2.10 (-4.59 to 8.79) | 14.12 (4.37 to 23.87) |
| Bosnia and Herzegovina | 12.05 (3.08 to 21.02) | 4.14 (0.42 to 7.86) | 3.15 (0.27 to 6.03) | 1.69 (-3.00 to 6.37) | 21.03 (11.19 to 30.86) |
| Botswana | 12.55 (1.22 to 23.88) | 2.66 (-0.45 to 5.77) | 1.87 (-0.66 to 4.40) | 1.09 (-3.09 to 5.27) | 18.18 (6.59 to 29.77) |
| Brazil | 12.04 (3.16 to 20.91) | 3.21 (0.55 to 5.86) | 2.57 (0.61 to 4.53) | 1.79 (-2.62 to 6.19) | 19.60 (9.59 to 29.60) |
| Brunei Darussalam | 13.52 (3.84 to 23.20) | 3.38 (0.35 to 6.40) | 2.34 (0.09 to 4.58) | 1.37 (-2.60 to 5.33) | 20.60 (10.28 to 30.91) |
| Bulgaria | 12.21 (1.51 to 22.91) | 3.80 (-0.25 to 7.84) | 2.87 (-0.45 to 6.20) | 1.54 (-3.54 to 6.62) | 20.42 (9.35 to 31.50) |
| Burkina Faso | 8.82 (0.81 to 16.83) | 2.32 (-0.46 to 5.11) | 1.72 (-0.84 to 4.28) | 0.84 (-3.67 to 5.35) | 13.70 (5.08 to 22.33) |
| Burundi | 9.38 (-1.04 to 19.80) | 2.50 (-1.02 to 6.02) | 2.01 (-1.26 to 5.28) | 1.29 (-4.17 to 6.75) | 15.18 (4.32 to 26.03) |
| Cabo Verde | 12.36 (3.86 to 20.87) | 3.42 (0.58 to 6.27) | 2.43 (0.36 to 4.51) | 1.32 (-2.45 to 5.09) | 19.55 (10.20 to 28.89) |
| Cambodia | 7.16 (0.38 to 13.94) | 2.21 (-0.50 to 4.93) | 1.83 (-0.91 to 4.57) | 1.19 (-3.79 to 6.17) | 12.40 (4.48 to 20.32) |
| Cameroon | 10.75 (1.90 to 19.59) | 2.94 (-0.17 to 6.05) | 2.17 (-0.42 to 4.77) | 1.21 (-3.19 to 5.61) | 17.07 (7.71 to 26.44) |
| Canada | 4.58 (1.58 to 7.59) | 2.37 (0.53 to 4.22) | 2.83 (0.95 to 4.70) | 2.69 (-3.66 to 9.04) | 12.47 (5.20 to 19.75) |
| Central African Republic | 12.17 (3.29 to 21.05) | 3.35 (0.20 to 6.50) | 2.43 (-0.11 to 4.96) | 1.47 (-3.07 to 6.00) | 19.42 (9.71 to 29.12) |
| Chad | 11.16 (0.21 to 22.11) | 2.59 (-0.92 to 6.11) | 1.80 (-1.21 to 4.81) | 0.86 (-4.10 to 5.82) | 16.41 (5.29 to 27.53) |
| Chile | 9.14 (2.75 to 15.52) | 3.42 (0.50 to 6.34) | 3.12 (0.63 to 5.62) | 2.33 (-3.38 to 8.05) | 18.02 (9.33 to 26.70) |
| China | 7.12 (2.34 to 11.90) | 2.60 (0.40 to 4.80) | 2.27 (0.36 to 4.19) | 1.57 (-2.66 to 5.80) | 13.56 (7.11 to 20.02) |
| Colombia | 7.80 (1.49 to 14.11) | 3.03 (0.19 to 5.88) | 2.90 (0.20 to 5.60) | 2.25 (-3.69 to 8.18) | 15.98 (7.47 to 24.49) |
| Comoros | 9.33 (0.34 to 18.32) | 2.52 (-0.52 to 5.56) | 2.03 (-0.72 to 4.78) | 1.31 (-3.51 to 6.14) | 15.19 (5.67 to 24.72) |
| Congo | 11.54 (0.19 to 22.90) | 2.82 (-1.08 to 6.73) | 2.04 (-1.40 to 5.48) | 1.19 (-4.21 to 6.59) | 17.60 (6.06 to 29.14) |
| Costa Rica | 10.40 (2.43 to 18.38) | 3.23 (0.24 to 6.22) | 2.71 (0.26 to 5.17) | 1.98 (-3.29 to 7.24) | 18.33 (8.97 to 27.69) |
| Cote d'Ivoire | 10.84 (0.59 to 21.09) | 2.77 (-0.78 to 6.32) | 1.98 (-1.18 to 5.14) | 1.01 (-4.13 to 6.14) | 16.60 (5.95 to 27.24) |
| Croatia | 13.46 (3.90 to 23.02) | 4.11 (0.52 to 7.69) | 2.97 (0.39 to 5.54) | 1.59 (-2.73 to 5.92) | 22.13 (11.75 to 32.50) |
| Cuba | 10.89 (1.04 to 20.74) | 3.00 (-0.35 to 6.36) | 2.41 (-0.44 to 5.26) | 1.60 (-3.32 to 6.51) | 17.90 (7.48 to 28.32) |
| Cyprus | 7.10 (0.37 to 13.82) | 3.16 (-0.21 to 6.52) | 3.20 (-0.18 to 6.59) | 2.52 (-4.29 to 9.32) | 15.97 (6.94 to 25.01) |
| Czech Republic | 10.72 (3.98 to 17.47) | 3.89 (0.87 to 6.90) | 3.15 (0.86 to 5.44) | 1.82 (-2.82 to 6.47) | 19.59 (11.05 to 28.12) |
| Denmark | 8.54 (2.06 to 15.01) | 2.92 (0.34 to 5.50) | 2.77 (0.55 to 4.99) | 2.05 (-3.07 to 7.17) | 16.28 (7.98 to 24.58) |
| Djibouti | 9.44 (-1.14 to 20.02) | 2.50 (-1.13 to 6.14) | 2.01 (-1.34 to 5.37) | 1.29 (-4.26 to 6.84) | 15.25 (4.29 to 26.21) |
| Dominican Republic | 13.84 (2.11 to 25.56) | 3.29 (-0.27 to 6.86) | 2.38 (-0.35 to 5.11) | 1.47 (-2.89 to 5.83) | 20.98 (9.03 to 32.94) |
| DR Congo | 9.87 (-0.24 to 19.98) | 2.62 (-1.02 to 6.27) | 2.00 (-1.35 to 5.34) | 1.22 (-4.38 to 6.82) | 15.71 (5.14 to 26.29) |
| Ecuador | 6.70 (1.56 to 11.85) | 2.40 (0.24 to 4.55) | 2.36 (0.30 to 4.42) | 2.06 (-3.17 to 7.29) | 13.52 (6.19 to 20.84) |
| Egypt | 10.85 (3.10 to 18.60) | 3.23 (0.55 to 5.92) | 2.47 (0.45 to 4.50) | 1.36 (-2.50 to 5.23) | 17.92 (9.17 to 26.67) |
| El Salvador | 8.84 (1.57 to 16.11) | 2.76 (-0.01 to 5.53) | 2.39 (-0.11 to 4.89) | 1.71 (-3.21 to 6.63) | 15.70 (7.18 to 24.21) |
| Equatorial Guinea | 11.05 (-0.91 to 23.02) | 2.78 (-1.30 to 6.86) | 2.04 (-1.61 to 5.70) | 1.21 (-4.61 to 7.03) | 17.08 (4.97 to 29.20) |
| Eritrea | 6.22 (-0.24 to 12.68) | 2.04 (-0.56 to 4.64) | 1.86 (-0.76 to 4.48) | 1.36 (-3.59 to 6.31) | 11.48 (3.90 to 19.06) |
| Estonia | 10.67 (2.13 to 19.21) | 3.29 (-0.04 to 6.62) | 2.52 (-0.27 to 5.31) | 1.10 (-3.26 to 5.47) | 17.58 (8.42 to 26.74) |
| Ethiopia | 7.49 (-0.30 to 15.27) | 1.82 (-0.55 to 4.19) | 1.47 (-0.71 to 3.64) | 0.86 (-3.19 to 4.92) | 11.64 (3.33 to 19.95) |
| Fiji | 10.76 (1.05 to 20.46) | 3.08 (-0.38 to 6.54) | 2.45 (-0.53 to 5.43) | 1.60 (-3.56 to 6.76) | 17.89 (7.53 to 28.25) |
| Finland | 8.80 (2.95 to 14.64) | 3.38 (0.64 to 6.11) | 3.14 (0.83 to 5.46) | 2.17 (-3.21 to 7.56) | 17.49 (9.32 to 25.67) |
| France | 6.39 (1.88 to 10.90) | 3.26 (0.58 to 5.94) | 3.42 (0.79 to 6.04) | 2.71 (-3.79 to 9.21) | 15.77 (7.65 to 23.89) |
| Gabon | 10.79 (0.33 to 21.25) | 2.70 (-0.89 to 6.30) | 1.99 (-1.18 to 5.17) | 1.18 (-4.10 to 6.46) | 16.66 (5.80 to 27.51) |
| Gambia | 11.12 (1.29 to 20.94) | 2.79 (-0.48 to 6.05) | 1.97 (-0.84 to 4.79) | 1.00 (-3.68 to 5.68) | 16.88 (6.66 to 27.10) |
| Georgia | 12.08 (3.28 to 20.88) | 3.62 (0.51 to 6.73) | 2.74 (0.38 to 5.10) | 1.51 (-2.69 to 5.71) | 19.95 (10.25 to 29.64) |
| Germany | 6.60 (1.43 to 11.77) | 3.11 (0.29 to 5.93) | 3.30 (0.50 to 6.09) | 2.66 (-3.80 to 9.12) | 15.67 (7.38 to 23.96) |
| Ghana | 10.13 (2.19 to 18.07) | 2.69 (-0.04 to 5.42) | 1.90 (-0.41 to 4.21) | 0.85 (-2.97 to 4.66) | 15.56 (7.08 to 24.03) |
| Greece | 7.04 (2.30 to 11.78) | 3.26 (0.66 to 5.86) | 3.38 (0.96 to 5.79) | 2.75 (-3.81 to 9.31) | 16.43 (8.05 to 24.81) |
| Grenada | 12.75 (1.56 to 23.95) | 3.19 (-0.26 to 6.65) | 2.42 (-0.39 to 5.23) | 1.55 (-3.13 to 6.22) | 19.91 (8.34 to 31.48) |
| Guatemala | 8.48 (1.27 to 15.70) | 2.86 (-0.07 to 5.78) | 2.56 (-0.13 to 5.26) | 1.85 (-3.45 to 7.15) | 15.75 (7.11 to 24.40) |
| Guinea | 11.89 (1.45 to 22.32) | 3.05 (-0.47 to 6.58) | 2.15 (-0.82 to 5.12) | 1.12 (-3.60 to 5.85) | 18.21 (7.46 to 28.97) |
| Guinea Bissau | 11.05 (-0.03 to 22.13) | 2.77 (-0.97 to 6.52) | 1.98 (-1.32 to 5.28) | 1.01 (-4.35 to 6.38) | 16.82 (5.51 to 28.13) |
| Guyana | 11.15 (2.45 to 19.85) | 3.26 (0.23 to 6.29) | 2.64 (0.13 to 5.14) | 1.79 (-3.12 to 6.70) | 18.84 (9.07 to 28.60) |
| Haiti | 12.07 (1.74 to 22.40) | 2.69 (0.04 to 5.34) | 2.05 (-0.05 to 4.16) | 1.36 (-2.72 to 5.44) | 18.17 (7.27 to 29.07) |
| Honduras | 8.93 (0.44 to 17.42) | 3.07 (-0.44 to 6.58) | 2.76 (-0.48 to 6.00) | 2.06 (-3.97 to 8.10) | 16.83 (6.97 to 26.68) |
| Hungary | 12.84 (1.60 to 24.09) | 3.75 (-0.19 to 7.69) | 2.79 (-0.41 to 5.99) | 1.50 (-3.42 to 6.42) | 20.88 (9.28 to 32.48) |
| Iceland | 5.62 (1.19 to 10.04) | 3.12 (0.36 to 5.88) | 3.60 (0.75 to 6.45) | 3.04 (-4.21 to 10.28) | 15.37 (6.79 to 23.95) |
| India | 8.57 (1.91 to 15.23) | 2.21 (0.14 to 4.29) | 1.79 (-0.01 to 3.59) | 1.44 (-2.50 to 5.38) | 14.02 (6.34 to 21.70) |
| Indonesia | 12.10 (3.15 to 21.05) | 2.84 (0.31 to 5.37) | 2.01 (0.11 to 3.91) | 1.25 (-2.30 to 4.79) | 18.19 (8.64 to 27.75) |
| Iran | 6.13 (2.58 to 9.68) | 3.10 (0.88 to 5.32) | 3.06 (1.17 to 4.94) | 2.03 (-2.81 to 6.86) | 14.30 (7.80 to 20.81) |
| Iraq | 13.72 (3.15 to 24.29) | 3.46 (0.27 to 6.66) | 2.38 (0.01 to 4.75) | 1.20 (-2.66 to 5.06) | 20.76 (9.78 to 31.74) |
| Ireland | 7.66 (0.61 to 14.70) | 2.75 (-0.12 to 5.61) | 2.70 (-0.04 to 5.45) | 2.14 (-3.66 to 7.94) | 15.24 (6.43 to 24.05) |
| Israel | 6.59 (1.82 to 11.36) | 3.13 (0.49 to 5.77) | 3.25 (0.75 to 5.75) | 2.56 (-3.65 to 8.76) | 15.53 (7.50 to 23.55) |
| Italy | 7.83 (2.91 to 12.75) | 3.57 (0.80 to 6.34) | 3.52 (1.14 to 5.90) | 2.64 (-3.60 to 8.87) | 17.55 (9.26 to 25.85) |
| Jamaica | 13.10 (1.88 to 24.32) | 3.36 (-0.23 to 6.95) | 2.51 (-0.24 to 5.26) | 1.58 (-2.98 to 6.15) | 20.55 (9.02 to 32.09) |
| Japan | 7.19 (2.92 to 11.46) | 3.00 (0.78 to 5.23) | 2.94 (1.05 to 4.83) | 2.28 (-3.11 to 7.67) | 15.42 (8.16 to 22.68) |
| Jordan | 10.00 (3.64 to 16.35) | 3.66 (0.88 to 6.45) | 2.97 (0.87 to 5.07) | 1.67 (-2.56 to 5.90) | 18.30 (10.33 to 26.27) |
| Kazakhstan | 11.65 (3.70 to 19.60) | 3.70 (0.75 to 6.66) | 2.85 (0.63 to 5.06) | 1.60 (-2.65 to 5.85) | 19.80 (10.63 to 28.97) |
| Kenya | 9.15 (1.26 to 17.04) | 2.44 (-0.10 to 4.98) | 1.94 (-0.31 to 4.20) | 1.22 (-3.07 to 5.51) | 14.76 (6.06 to 23.46) |
| Kiribati | 11.02 (-0.68 to 22.72) | 2.09 (-0.63 to 4.81) | 1.59 (-0.75 to 3.94) | 0.97 (-3.21 to 5.15) | 15.68 (3.72 to 27.64) |
| Kuwait | 10.14 (2.74 to 17.53) | 3.46 (0.46 to 6.46) | 2.84 (0.37 to 5.31) | 1.68 (-2.96 to 6.33) | 18.12 (9.40 to 26.84) |
| Kyrgyzstan | 11.62 (2.95 to 20.29) | 3.63 (0.41 to 6.86) | 2.77 (0.20 to 5.34) | 1.56 (-2.92 to 6.03) | 19.58 (9.97 to 29.18) |
| Lao PDR | 8.44 (0.81 to 16.07) | 2.27 (-0.49 to 5.04) | 1.75 (-0.81 to 4.31) | 1.05 (-3.61 to 5.71) | 13.51 (5.06 to 21.97) |
| Latvia | 11.86 (2.51 to 21.20) | 3.79 (0.11 to 7.47) | 2.85 (-0.14 to 5.84) | 1.39 (-3.21 to 5.99) | 19.89 (9.88 to 29.90) |
| Lebanon | 9.97 (2.49 to 17.44) | 3.19 (0.43 to 5.95) | 2.58 (0.29 to 4.87) | 1.49 (-2.75 to 5.73) | 17.22 (8.68 to 25.75) |
| Lesotho | 11.84 (2.30 to 21.38) | 2.96 (0.01 to 5.90) | 2.16 (-0.29 to 4.62) | 1.34 (-3.00 to 5.67) | 18.29 (8.15 to 28.44) |
| Liberia | 11.57 (1.54 to 21.59) | 2.84 (-0.40 to 6.08) | 2.01 (-0.78 to 4.79) | 1.02 (-3.55 to 5.59) | 17.44 (6.99 to 27.89) |
| Libya | 11.40 (2.60 to 20.19) | 3.93 (0.30 to 7.57) | 3.05 (0.12 to 5.97) | 1.70 (-3.18 to 6.58) | 20.07 (10.39 to 29.76) |
| Lithuania | 13.19 (3.02 to 23.36) | 3.86 (0.19 to 7.53) | 2.71 (-0.18 to 5.61) | 1.22 (-3.04 to 5.48) | 20.98 (10.38 to 31.59) |
| Luxembourg | 6.64 (2.07 to 11.22) | 3.03 (0.56 to 5.50) | 3.26 (0.91 to 5.61) | 2.73 (-3.81 to 9.27) | 15.66 (7.49 to 23.82) |
| Macedonia (TFYR) | 12.20 (1.68 to 22.72) | 3.80 (-0.16 to 7.75) | 2.88 (-0.41 to 6.17) | 1.55 (-3.57 to 6.66) | 20.42 (9.43 to 31.41) |
| Madagascar | 10.17 (-0.98 to 21.33) | 2.35 (-1.11 to 5.80) | 1.80 (-1.29 to 4.89) | 1.08 (-4.04 to 6.20) | 15.40 (4.03 to 26.77) |
| Malawi | 7.75 (2.00 to 13.50) | 2.84 (0.30 to 5.38) | 2.60 (0.40 to 4.80) | 2.01 (-3.14 to 7.17) | 15.20 (7.49 to 22.92) |
| Malaysia | 11.94 (3.46 to 20.42) | 3.34 (0.29 to 6.39) | 2.42 (0.07 to 4.78) | 1.43 (-2.75 to 5.62) | 19.13 (9.87 to 28.39) |
| Maldives | 9.67 (1.70 to 17.64) | 3.30 (-0.13 to 6.73) | 2.67 (-0.40 to 5.74) | 1.75 (-3.75 to 7.25) | 17.39 (8.25 to 26.54) |
| Mali | 10.24 (0.55 to 19.94) | 2.75 (-0.75 to 6.25) | 2.04 (-0.98 to 5.07) | 1.09 (-3.97 to 6.16) | 16.13 (6.02 to 26.25) |
| Malta | 6.52 (1.76 to 11.28) | 3.17 (0.46 to 5.87) | 3.34 (0.70 to 5.97) | 2.68 (-3.93 to 9.29) | 15.70 (7.44 to 23.96) |
| Mauritania | 11.06 (-0.17 to 22.29) | 2.77 (-1.02 to 6.55) | 1.97 (-1.36 to 5.30) | 1.01 (-4.33 to 6.34) | 16.81 (5.38 to 28.24) |
| Mauritius | 8.47 (2.59 to 14.36) | 3.36 (0.57 to 6.15) | 3.05 (0.63 to 5.47) | 2.19 (-3.25 to 7.63) | 17.07 (8.94 to 25.20) |
| Mexico | 8.39 (2.59 to 14.19) | 2.79 (0.53 to 5.05) | 2.51 (0.61 to 4.42) | 1.92 (-2.81 to 6.65) | 15.62 (7.96 to 23.28) |
| Micronesia (Federated States of) | 8.86 (1.25 to 16.47) | 2.66 (-0.08 to 5.39) | 2.31 (-0.22 to 4.84) | 1.65 (-3.32 to 6.63) | 15.47 (6.70 to 24.24) |
| Moldova | 13.82 (3.34 to 24.29) | 3.65 (0.18 to 7.13) | 2.46 (-0.11 to 5.04) | 1.06 (-2.73 to 4.85) | 20.99 (10.19 to 31.80) |
| Mongolia | 11.84 (3.42 to 20.27) | 3.27 (0.60 to 5.94) | 2.44 (0.46 to 4.42) | 1.33 (-2.41 to 5.07) | 18.88 (9.56 to 28.21) |
| Montenegro | 12.23 (1.38 to 23.08) | 3.80 (-0.26 to 7.85) | 2.87 (-0.48 to 6.22) | 1.54 (-3.38 to 6.46) | 20.44 (9.29 to 31.59) |
| Morocco | 9.60 (2.51 to 16.68) | 3.22 (0.44 to 6.00) | 2.64 (0.35 to 4.92) | 1.55 (-2.82 to 5.92) | 17.01 (8.66 to 25.35) |
| Mozambique | 10.69 (0.64 to 20.74) | 2.33 (-0.39 to 5.04) | 1.73 (-0.63 to 4.08) | 1.01 (-3.17 to 5.19) | 15.75 (5.29 to 26.21) |
| Myanmar | 11.13 (1.71 to 20.55) | 2.47 (-0.26 to 5.20) | 1.71 (-0.59 to 4.01) | 0.91 (-2.94 to 4.76) | 16.22 (6.47 to 25.97) |
| Namibia | 12.23 (0.52 to 23.95) | 2.43 (-0.66 to 5.52) | 1.69 (-0.95 to 4.33) | 0.95 (-3.45 to 5.34) | 17.30 (5.38 to 29.23) |
| Nepal | 10.21 (1.78 to 18.64) | 1.95 (0.14 to 3.76) | 1.31 (-0.07 to 2.69) | 0.79 (-1.85 to 3.44) | 14.26 (5.46 to 23.06) |
| Netherlands | 6.66 (1.78 to 11.53) | 3.08 (0.48 to 5.68) | 3.32 (0.83 to 5.80) | 2.75 (-3.79 to 9.30) | 15.81 (7.50 to 24.12) |
| New Zealand | 7.41 (1.82 to 12.99) | 2.72 (0.41 to 5.03) | 2.72 (0.60 to 4.84) | 2.17 (-3.18 to 7.51) | 15.02 (7.16 to 22.88) |
| Nicaragua | 9.77 (0.16 to 19.38) | 2.90 (-0.70 to 6.51) | 2.45 (-0.83 to 5.73) | 1.75 (-3.96 to 7.46) | 16.88 (6.37 to 27.39) |
| Niger | 12.00 (0.38 to 23.62) | 2.57 (-0.85 to 5.98) | 1.72 (-1.21 to 4.65) | 0.77 (-3.90 to 5.44) | 17.06 (5.28 to 28.84) |
| Nigeria | 10.96 (3.12 to 18.79) | 2.83 (0.33 to 5.33) | 1.95 (0.06 to 3.84) | 0.87 (-2.30 to 4.05) | 16.61 (8.19 to 25.03) |
| North Korea | 7.06 (0.40 to 13.72) | 2.54 (-0.46 to 5.54) | 2.28 (-0.70 to 5.26) | 1.65 (-3.93 to 7.22) | 13.53 (5.43 to 21.63) |
| Norway | 6.78 (1.09 to 12.47) | 3.04 (0.11 to 5.96) | 3.23 (0.31 to 6.15) | 2.65 (-3.98 to 9.27) | 15.69 (7.11 to 24.28) |
| Occupied Palestinian Territory | 10.48 (2.35 to 18.61) | 3.69 (0.25 to 7.13) | 2.96 (0.10 to 5.82) | 1.72 (-3.24 to 6.67) | 18.84 (9.55 to 28.13) |
| Oman | 11.60 (2.93 to 20.28) | 3.26 (0.40 to 6.13) | 2.44 (0.24 to 4.64) | 1.30 (-2.54 to 5.13) | 18.61 (9.17 to 28.05) |
| Pakistan | 12.09 (0.05 to 24.13) | 1.97 (-1.00 to 4.95) | 1.28 (-1.21 to 3.77) | 0.77 (-3.41 to 4.95) | 16.11 (3.96 to 28.26) |
| Panama | 9.55 (0.91 to 18.19) | 3.00 (-0.34 to 6.34) | 2.60 (-0.38 to 5.57) | 1.89 (-3.57 to 7.35) | 17.04 (7.25 to 26.82) |
| Papua New Guinea | 7.63 (-1.11 to 16.36) | 2.18 (-1.01 to 5.36) | 1.86 (-1.29 to 5.00) | 1.29 (-4.29 to 6.87) | 12.95 (3.33 to 22.56) |
| Paraguay | 15.05 (1.91 to 28.19) | 3.19 (-0.34 to 6.72) | 2.25 (-0.41 to 4.90) | 1.38 (-2.81 to 5.57) | 21.87 (8.62 to 35.13) |
| Peru | 4.64 (1.49 to 7.78) | 1.91 (0.42 to 3.40) | 2.13 (0.68 to 3.57) | 2.21 (-3.04 to 7.46) | 10.88 (4.61 to 17.15) |
| Philippines | 9.71 (2.00 to 17.43) | 2.45 (-0.05 to 4.94) | 1.77 (-0.34 to 3.89) | 0.96 (-2.62 to 4.54) | 14.89 (6.65 to 23.13) |
| Poland | 13.13 (4.14 to 22.11) | 3.84 (0.80 to 6.88) | 2.82 (0.69 to 4.95) | 1.47 (-2.41 to 5.34) | 21.26 (11.33 to 31.18) |
| Portugal | 7.55 (2.20 to 12.90) | 3.47 (0.56 to 6.39) | 3.43 (0.75 to 6.11) | 2.58 (-3.74 to 8.90) | 17.04 (8.62 to 25.46) |
| Puerto Rico | 11.75 (0.00 to 23.50) | 3.16 (-0.84 to 7.15) | 2.49 (-0.95 to 5.93) | 1.65 (-3.89 to 7.19) | 19.05 (7.02 to 31.08) |
| Qatar | 10.51 (2.48 to 18.54) | 3.75 (0.39 to 7.10) | 3.01 (0.25 to 5.77) | 1.73 (-3.15 to 6.62) | 19.00 (9.79 to 28.22) |
| Romania | 13.44 (4.06 to 22.81) | 3.76 (0.60 to 6.92) | 2.67 (0.41 to 4.93) | 1.35 (-2.40 to 5.09) | 21.21 (11.14 to 31.28) |
| Russian Federation | 12.41 (4.27 to 20.56) | 3.88 (0.72 to 7.04) | 2.77 (0.55 to 4.99) | 1.23 (-2.40 to 4.87) | 20.30 (11.22 to 29.37) |
| Rwanda | 8.07 (0.01 to 16.13) | 2.24 (-0.58 to 5.06) | 1.86 (-0.78 to 4.51) | 1.22 (-3.66 to 6.09) | 13.39 (4.53 to 22.25) |
| Saint Lucia | 10.93 (1.51 to 20.35) | 3.22 (-0.15 to 6.59) | 2.61 (-0.24 to 5.46) | 1.75 (-3.30 to 6.79) | 18.50 (8.22 to 28.78) |
| Saint Vincent and the Grenadines | 10.98 (2.41 to 19.55) | 3.45 (0.20 to 6.70) | 2.82 (0.14 to 5.50) | 1.91 (-3.20 to 7.01) | 19.15 (9.41 to 28.90) |
| Samoa | 10.16 (0.59 to 19.73) | 2.60 (-0.29 to 5.49) | 2.15 (-0.42 to 4.72) | 1.46 (-3.30 to 6.23) | 16.38 (6.01 to 26.75) |
| Sao Tome and Principe | 13.33 (3.14 to 23.52) | 2.88 (0.21 to 5.54) | 1.90 (-0.07 to 3.87) | 0.91 (-2.41 to 4.24) | 19.02 (8.44 to 29.60) |
| Saudi Arabia | 8.54 (1.90 to 15.17) | 3.13 (0.25 to 6.00) | 2.69 (0.11 to 5.28) | 1.63 (-3.23 to 6.49) | 15.99 (7.85 to 24.13) |
| Senegal | 11.83 (1.39 to 22.27) | 2.86 (-0.53 to 6.26) | 2.01 (-0.81 to 4.84) | 1.07 (-3.52 to 5.66) | 17.78 (6.97 to 28.60) |
| Serbia | 12.53 (3.79 to 21.28) | 4.11 (0.64 to 7.58) | 3.04 (0.42 to 5.67) | 1.56 (-2.69 to 5.81) | 21.25 (11.58 to 30.92) |
| Seychelles | 12.10 (2.75 to 21.45) | 3.48 (0.21 to 6.74) | 2.63 (0.09 to 5.17) | 1.61 (-2.93 to 6.16) | 19.82 (9.65 to 30.00) |
| Sierra Leone | 12.01 (0.95 to 23.07) | 2.62 (-0.72 to 5.97) | 1.76 (-1.11 to 4.62) | 0.79 (-3.79 to 5.37) | 17.18 (5.94 to 28.43) |
| Singapore | 7.03 (2.47 to 11.60) | 3.36 (0.77 to 5.94) | 3.47 (1.10 to 5.83) | 2.83 (-3.80 to 9.47) | 16.69 (8.30 to 25.07) |
| Slovakia | 11.42 (3.08 to 19.77) | 3.99 (0.46 to 7.52) | 3.13 (0.27 to 5.99) | 1.74 (-3.18 to 6.67) | 20.29 (10.80 to 29.78) |
| Slovenia | 12.20 (1.42 to 22.98) | 3.79 (-0.27 to 7.84) | 2.87 (-0.45 to 6.18) | 1.54 (-3.53 to 6.61) | 20.40 (9.23 to 31.57) |
| Solomon Islands | 8.38 (1.35 to 15.40) | 2.79 (-0.04 to 5.62) | 2.42 (-0.19 to 5.02) | 1.71 (-3.49 to 6.90) | 15.29 (6.85 to 23.74) |
| Somalia | 10.06 (0.24 to 19.88) | 2.56 (-0.58 to 5.70) | 2.00 (-0.83 to 4.84) | 1.26 (-3.65 to 6.16) | 15.88 (5.50 to 26.25) |
| South Africa | 12.43 (3.16 to 21.69) | 2.92 (0.42 to 5.41) | 2.12 (0.25 to 3.98) | 1.35 (-2.34 to 5.04) | 18.81 (8.84 to 28.78) |
| South Korea | 5.43 (2.03 to 8.83) | 2.73 (0.69 to 4.76) | 3.12 (1.15 to 5.08) | 2.73 (-3.62 to 9.09) | 14.00 (6.44 to 21.57) |
| South Sudan | 9.43 (-0.89 to 19.75) | 2.51 (-0.99 to 6.01) | 2.02 (-1.31 to 5.35) | 1.30 (-4.31 to 6.91) | 15.26 (4.41 to 26.11) |
| Spain | 5.30 (1.70 to 8.89) | 2.97 (0.56 to 5.37) | 3.47 (1.07 to 5.87) | 2.93 (-3.94 to 9.81) | 14.67 (6.63 to 22.70) |
| Sri Lanka | 10.13 (1.79 to 18.47) | 2.67 (-0.13 to 5.46) | 2.08 (-0.39 to 4.55) | 1.52 (-3.13 to 6.17) | 16.39 (7.15 to 25.63) |
| Sudan | 11.25 (0.58 to 21.92) | 2.28 (-0.39 to 4.95) | 1.66 (-0.60 to 3.91) | 0.93 (-2.90 to 4.76) | 16.11 (5.20 to 27.03) |
| Suriname | 11.92 (1.73 to 22.11) | 3.31 (-0.21 to 6.82) | 2.60 (-0.25 to 5.44) | 1.73 (-3.32 to 6.78) | 19.56 (8.63 to 30.48) |
| Swaziland | 12.47 (2.17 to 22.76) | 2.98 (-0.12 to 6.07) | 2.13 (-0.41 to 4.67) | 1.30 (-3.07 to 5.67) | 18.88 (8.12 to 29.64) |
| Sweden | 6.69 (1.99 to 11.40) | 3.04 (0.52 to 5.55) | 3.21 (0.81 to 5.61) | 2.59 (-3.66 to 8.84) | 15.53 (7.51 to 23.55) |
| Switzerland | 4.08 (0.92 to 7.24) | 2.40 (0.29 to 4.50) | 3.01 (0.67 to 5.36) | 2.92 (-4.03 to 9.88) | 12.41 (4.64 to 20.18) |
| Syrian Arab Republic | 11.23 (1.88 to 20.58) | 3.65 (-0.04 to 7.33) | 2.82 (-0.24 to 5.88) | 1.58 (-3.45 to 6.61) | 19.27 (9.14 to 29.40) |
| Taiwan | 5.51 (1.25 to 9.76) | 2.54 (0.14 to 4.94) | 2.65 (0.24 to 5.05) | 2.24 (-3.60 to 8.09) | 12.94 (5.71 to 20.17) |
| Tajikistan | 12.82 (3.82 to 21.83) | 3.65 (0.67 to 6.62) | 2.66 (0.43 to 4.90) | 1.43 (-2.50 to 5.36) | 20.57 (10.69 to 30.45) |
| Tanzania | 9.37 (1.18 to 17.55) | 2.45 (-0.15 to 5.05) | 1.90 (-0.40 to 4.19) | 1.03 (-2.93 to 4.98) | 14.74 (6.04 to 23.44) |
| Thailand | 8.20 (1.35 to 15.06) | 2.69 (-0.26 to 5.64) | 2.22 (-0.54 to 4.97) | 1.49 (-3.62 to 6.60) | 14.60 (6.53 to 22.66) |
| Timor-Leste | 10.06 (-0.35 to 20.48) | 1.82 (-0.87 to 4.52) | 1.17 (-1.28 to 3.62) | 0.45 (-3.78 to 4.67) | 13.50 (2.89 to 24.12) |
| Togo | 10.45 (0.71 to 20.20) | 2.55 (-0.62 to 5.71) | 1.80 (-1.00 to 4.61) | 0.87 (-3.83 to 5.57) | 15.67 (5.54 to 25.81) |
| Tonga | 12.03 (2.18 to 21.88) | 3.00 (0.16 to 5.84) | 2.32 (0.15 to 4.50) | 1.48 (-2.66 to 5.63) | 18.84 (8.33 to 29.35) |
| Trinidad and Tobago | 11.59 (1.20 to 21.98) | 3.07 (-0.34 to 6.47) | 2.43 (-0.42 to 5.28) | 1.65 (-3.35 to 6.64) | 18.74 (7.79 to 29.68) |
| Tunisia | 9.21 (2.26 to 16.17) | 3.46 (0.33 to 6.60) | 2.93 (0.28 to 5.58) | 1.79 (-3.15 to 6.74) | 17.40 (9.00 to 25.80) |
| Turkey | 8.74 (3.05 to 14.43) | 3.55 (0.72 to 6.38) | 3.03 (0.74 to 5.33) | 1.86 (-2.93 to 6.65) | 17.18 (9.45 to 24.91) |
| Turkmenistan | 10.85 (3.30 to 18.39) | 3.43 (0.64 to 6.23) | 2.71 (0.57 to 4.86) | 1.56 (-2.79 to 5.92) | 18.56 (9.74 to 27.37) |
| Uganda | 8.76 (-0.54 to 18.06) | 1.89 (-0.66 to 4.44) | 1.42 (-0.94 to 3.78) | 0.73 (-3.37 to 4.83) | 12.80 (3.11 to 22.49) |
| Ukraine | 12.25 (4.29 to 20.20) | 3.87 (0.80 to 6.94) | 2.80 (0.62 to 4.98) | 1.32 (-2.38 to 5.02) | 20.24 (11.30 to 29.17) |
| United Arab Emirates | 10.30 (2.69 to 17.90) | 3.16 (0.43 to 5.90) | 2.51 (0.30 to 4.72) | 1.43 (-2.71 to 5.57) | 17.40 (8.73 to 26.07) |
| United Kingdom | 5.97 (2.23 to 9.71) | 2.58 (0.68 to 4.48) | 2.79 (1.05 to 4.53) | 2.36 (-3.17 to 7.90) | 13.70 (6.69 to 20.72) |
| United States of America | 7.58 (2.30 to 12.86) | 2.91 (0.61 to 5.21) | 2.91 (0.90 to 4.93) | 2.31 (-3.17 to 7.79) | 15.72 (7.86 to 23.57) |
| Uruguay | 11.24 (2.74 to 19.74) | 3.57 (0.33 to 6.81) | 2.95 (0.29 to 5.61) | 2.00 (-3.19 to 7.19) | 19.76 (9.95 to 29.57) |
| Uzbekistan | 12.62 (2.93 to 22.32) | 3.07 (0.46 to 5.69) | 2.20 (0.31 to 4.08) | 1.10 (-2.31 to 4.51) | 18.99 (8.68 to 29.30) |
| Vanuatu | 11.07 (0.41 to 21.73) | 2.75 (-0.64 to 6.14) | 2.13 (-0.85 to 5.11) | 1.37 (-3.65 to 6.39) | 17.32 (6.17 to 28.46) |
| Venezuela | 10.66 (2.56 to 18.75) | 3.25 (0.34 to 6.15) | 2.73 (0.31 to 5.16) | 1.99 (-3.08 to 7.06) | 18.63 (9.15 to 28.10) |
| Vietnam | 7.80 (1.98 to 13.62) | 2.75 (0.22 to 5.28) | 2.39 (0.09 to 4.69) | 1.72 (-3.11 to 6.54) | 14.66 (7.25 to 22.07) |
| Yemen | 8.03 (0.10 to 15.97) | 2.47 (-0.53 to 5.46) | 2.01 (-0.75 to 4.78) | 1.07 (-3.74 to 5.88) | 13.59 (4.95 to 22.22) |
| Zambia | 8.73 (1.58 to 15.88) | 2.61 (0.10 to 5.12) | 2.19 (-0.04 to 4.41) | 1.46 (-3.00 to 5.92) | 14.99 (6.72 to 23.25) |
| Zimbabwe | 12.13 (-0.35 to 24.61) | 2.75 (-1.02 to 6.53) | 1.98 (-1.28 to 5.25) | 1.20 (-3.97 to 6.36) | 18.06 (5.48 to 30.65) |

**Supplementary Table 6 – Number of dementia cases per country attributable to hypertension (HTN) total and by age range.**

| **Country** | **30-44 (95% CI)** | **45-54 (95% CI)** | **55-64 (95% CI)** | **65-74 (95% CI)** | **Total (95% CI)** |
| --- | --- | --- | --- | --- | --- |
| **Afghanistan** | 6 606 (787 to 12426) | 1 215 (-189 to 2618) | 826 (-327 to 1979) | 545 (-1553 to 2643) | 9 193 (3179 to 15206) |
| **Albania** | 4 211 (1268 to 7154) | 1 280 (210 to 2351) | 963 (122 to 1805) | 506 (-985 to 1997) | 6 961 (3652 to 10269) |
| **Algeria** | 24 814 (7320 to 42307) | 8 786 (1467 to 16104) | 7 166 (1242 to 13090) | 4 221 (-7182 to 15625) | 44 986 (23864 to 66108) |
| **Angola** | 5 258 (274 to 10243) | 1 226 (-394 to 2846) | 873 (-580 to 2325) | 494 (-1868 to 2855) | 7 851 (2698 to 13005) |
| **Antigua and Barbuda** | 70 (0 to 139) | 19 (-4 to 42) | 15 (-5 to 35) | 10 (-22 to 42) | 113 (42 to 184) |
| **Argentina** | 51 975 (12488 to 91461) | 12 984 (1706 to 24263) | 10 054 (1581 to 18526) | 6 775 (-10686 to 24237) | 81 788 (38602 to 124975) |
| **Armenia** | 4 239 (1069 to 7410) | 1 132 (134 to 2130) | 803 (77 to 1529) | 417 (-818 to 1652) | 6 592 (3229 to 9955) |
| **Australia** | 23 888 (6942 to 40835) | 9 541 (1720 to 17362) | 9 905 (2737 to 17073) | 8 275 (-11570 to 28119) | 51 609 (24831 to 78387) |
| **Austria** | 11 990 (782 to 23198) | 4 638 (-216 to 9491) | 4 504 (-175 to 9183) | 3 417 (-5782 to 12616) | 24 549 (10784 to 38313) |
| **Azerbaijan** | 5 965 (1570 to 10360) | 1 796 (244 to 3348) | 1 384 (191 to 2577) | 787 (-1449 to 3023) | 9 933 (5014 to 14851) |
| **Bahamas** | 233 (13 to 452) | 53 (-10 to 116) | 40 (-13 to 94) | 26 (-64 to 117) | 352 (126 to 578) |
| **Bahrain** | 514 (43 to 986) | 170 (-14 to 353) | 137 (-22 to 296) | 79 (-187 to 345) | 900 (390 to 1410) |
| **Bangladesh** | 50 222 (8398 to 92046) | 9 969 (-174 to 20112) | 6 833 (-1663 to 15329) | 4 479 (-11753 to 20710) | 71 503 (27449 to 115557) |
| **Barbados** | 353 (69 to 638) | 109 (2 to 216) | 87 (1 to 173) | 58 (-99 to 215) | 607 (296 to 918) |
| **Belarus** | 19 301 (6676 to 31927) | 5 853 (1209 to 10497) | 3 940 (816 to 7064) | 1 770 (-3182 to 6721) | 30 864 (17212 to 44516) |
| **Belgium** | 13 051 (4108 to 21993) | 5 816 (1129 to 10502) | 6 070 (1736 to 10405) | 4 924 (-6717 to 16565) | 29 861 (14713 to 45008) |
| **Belize** | 141 (-2 to 285) | 38 (-10 to 86) | 31 (-12 to 74) | 22 (-52 to 95) | 232 (81 to 383) |
| **Benin** | 1 553 (445 to 2660) | 482 (67 to 896) | 378 (26 to 730) | 212 (-450 to 875) | 2 625 (1329 to 3921) |
| **Bhutan** | 317 (52 to 582) | 58 (1 to 115) | 39 (-6 to 84) | 26 (-59 to 111) | 440 (166 to 715) |
| **Bolivia** | 3 103 (-477 to 6682) | 1 097 (-416 to 2610) | 1 069 (-482 to 2620) | 920 (-2012 to 3851) | 6 189 (1915 to 10463) |
| **Bosnia and Herzegovina** | 5 793 (1480 to 10105) | 1 992 (204 to 3779) | 1 514 (128 to 2900) | 811 (-1442 to 3064) | 10 110 (5381 to 14838) |
| **Botswana** | 846 (83 to 1609) | 179 (-30 to 389) | 126 (-45 to 296) | 73 (-208 to 355) | 1 225 (444 to 2005) |
| **Brazil** | 222 666 (58467 to 386864) | 59 324 (10201 to 108448) | 47 489 (11262 to 83716) | 33 032 (-48466 to 114530) | 362 511 (177346 to 547675) |
| **Brunei Darussalam** | 213 (60 to 365) | 53 (6 to 101) | 37 (1 to 72) | 21 (-41 to 84) | 324 (162 to 487) |
| **Bulgaria** | 16 522 (2047 to 30997) | 5 136 (-336 to 10608) | 3 886 (-613 to 8385) | 2 088 (-4786 to 8961) | 27 632 (12650 to 42613) |
| **Burkina Faso** | 2 980 (274 to 5687) | 785 (-156 to 1726) | 581 (-283 to 1445) | 284 (-1241 to 1809) | 4 630 (1716 to 7545) |
| **Burundi** | 1 388 (-153 to 2928) | 369 (-151 to 890) | 297 (-186 to 781) | 190 (-618 to 998) | 2 245 (640 to 3850) |
| **Cabo Verde** | 315 (98 to 531) | 87 (15 to 160) | 62 (9 to 115) | 34 (-62 to 130) | 498 (260 to 736) |
| **Cambodia** | 3 956 (212 to 7700) | 1 223 (-277 to 2723) | 1 011 (-500 to 2522) | 657 (-2093 to 3406) | 6 847 (2473 to 11220) |
| **Cameroon** | 4 425 (782 to 8069) | 1 212 (-68 to 2492) | 895 (-174 to 1965) | 498 (-1315 to 2312) | 7 031 (3174 to 10888) |
| **Canada** | 26 918 (9275 to 44561) | 13 937 (3084 to 24790) | 16 601 (5609 to 27592) | 15 805 (-21496 to 53105) | 73 260 (30524 to 115997) |
| **Central African Republic** | 999 (270 to 1727) | 275 (16 to 533) | 199 (-9 to 407) | 120 (-252 to 493) | 1 593 (797 to 2390) |
| **Chad** | 2 246 (42 to 4451) | 522 (-186 to 1231) | 363 (-244 to 969) | 173 (-826 to 1171) | 3 304 (1066 to 5543) |
| **Chile** | 15 982 (4810 to 27154) | 5 986 (875 to 11098) | 5 461 (1099 to 9822) | 4 084 (-5913 to 14081) | 31 513 (16328 to 46699) |
| **China** | 1 091 490 (358490 to 1824490) | 398 567 (61662 to 735471) | 348 635 (54422 to 642849) | 240 234 (-408244 to 888713) | 2 078 926 (1089467 to 3068385) |
| **Colombia** | 28 816 (5518 to 52115) | 11 208 (686 to 21731) | 10 712 (745 to 20680) | 8 296 (-13622 to 30213) | 59 032 (27589 to 90475) |
| **Comoros** | 193 (7 to 380) | 52 (-11 to 115) | 42 (-15 to 99) | 27 (-73 to 127) | 315 (118 to 513) |
| **Congo** | 1 332 (22 to 2642) | 326 (-125 to 776) | 235 (-161 to 632) | 138 (-485 to 761) | 2 031 (699 to 3362) |
| **Costa Rica** | 3 396 (793 to 5999) | 1 054 (79 to 2029) | 886 (83 to 1689) | 646 (-1072 to 2364) | 5 982 (2928 to 9036) |
| **Cote d'Ivoire** | 3 734 (205 to 7263) | 953 (-270 to 2177) | 681 (-408 to 1771) | 347 (-1422 to 2115) | 5 715 (2050 to 9380) |
| **Croatia** | 11 232 (3256 to 19207) | 3 426 (437 to 6415) | 2 474 (322 to 4626) | 1 329 (-2281 to 4938) | 18 460 (9807 to 27113) |
| **Cuba** | 12 641 (1209 to 24072) | 3 485 (-412 to 7382) | 2 797 (-509 to 6103) | 1 851 (-3849 to 7552) | 20 774 (8676 to 32872) |
| **Cyprus** | 1 004 (52 to 1955) | 446 (-29 to 922) | 453 (-25 to 932) | 356 (-606 to 1318) | 2 259 (981 to 3537) |
| **Czech Republic** | 20 666 (7668 to 33665) | 7 490 (1674 to 13305) | 6 078 (1663 to 10493) | 3 517 (-5438 to 12471) | 37 751 (21301 to 54201) |
| **Denmark** | 6 994 (1692 to 12297) | 2 393 (282 to 4503) | 2 270 (451 to 4089) | 1 679 (-2517 to 5875) | 13 336 (6536 to 20135) |
| **Djibouti** | 197 (-24 to 417) | 52 (-24 to 128) | 42 (-28 to 112) | 27 (-89 to 143) | 318 (89 to 547) |
| **Dominican Republic** | 7 159 (1093 to 13224) | 1 704 (-140 to 3549) | 1 231 (-182 to 2644) | 762 (-1493 to 3017) | 10 856 (4670 to 17042) |
| **DR Congo** | 15 175 (-362 to 30711) | 4 033 (-1569 to 9634) | 3 067 (-2074 to 8209) | 1 874 (-6734 to 10483) | 24 149 (7893 to 40405) |
| **Ecuador** | 5 883 (1366 to 10401) | 2 106 (214 to 3998) | 2 067 (259 to 3875) | 1 805 (-2786 to 6396) | 11 862 (5435 to 18289) |
| **Egypt** | 33 172 (9487 to 56858) | 9 884 (1682 to 18086) | 7 562 (1372 to 13752) | 4 165 (-7644 to 15975) | 54 784 (28042 to 81527) |
| **El Salvador** | 3 241 (574 to 5908) | 1 012 (-5 to 2028) | 876 (-42 to 1795) | 628 (-1176 to 2432) | 5 757 (2635 to 8879) |
| **Equatorial Guinea** | 267 (-22 to 556) | 67 (-32 to 166) | 49 (-39 to 138) | 29 (-111 to 170) | 413 (120 to 705) |
| **Eritrea** | 477 (-19 to 972) | 156 (-43 to 356) | 143 (-58 to 344) | 104 (-275 to 484) | 880 (299 to 1461) |
| **Estonia** | 2 849 (569 to 5130) | 879 (-11 to 1769) | 674 (-71 to 1419) | 294 (-871 to 1460) | 4 696 (2250 to 7142) |
| **Ethiopia** | 13 028 (-514 to 26571) | 3 166 (-954 to 7287) | 2 555 (-1228 to 6338) | 1 502 (-5559 to 8562) | 20 251 (5791 to 34711) |
| **Fiji** | 336 (33 to 639) | 96 (-12 to 204) | 77 (-17 to 170) | 50 (-111 to 211) | 559 (235 to 883) |
| **Finland** | 8 583 (2880 to 14285) | 3 296 (629 to 5964) | 3 066 (806 to 5326) | 2 121 (-3128 to 7370) | 17 066 (9087 to 25044) |
| **France** | 76 895 (22614 to 131175) | 39 223 (6985 to 71461) | 41 098 (9511 to 72685) | 32 576 (-45649 to 110801) | 189 791 (92036 to 287547) |
| **Gabon** | 625 (19 to 1232) | 157 (-52 to 365) | 115 (-69 to 300) | 68 (-238 to 374) | 966 (336 to 1595) |
| **Gambia** | 422 (49 to 795) | 106 (-18 to 230) | 75 (-32 to 182) | 38 (-140 to 216) | 641 (253 to 1029) |
| **Georgia** | 5 593 (1520 to 9667) | 1 676 (236 to 3115) | 1 267 (175 to 2359) | 697 (-1246 to 2641) | 9 234 (4747 to 13720) |
| **Germany** | 111 579 (24173 to 198985) | 52 603 (4910 to 100296) | 55 786 (8521 to 103050) | 45 025 (-64182 to 154232) | 264 993 (124813 to 405173) |
| **Ghana** | 5 800 (1253 to 10347) | 1 539 (-25 to 3104) | 1 087 (-236 to 2410) | 484 (-1699 to 2667) | 8 911 (4056 to 13766) |
| **Greece** | 14 537 (4755 to 24320) | 6 733 (1366 to 12100) | 6 970 (1990 to 11951) | 5 674 (-7861 to 19208) | 33 914 (16620 to 51208) |
| **Grenada** | 135 (16 to 253) | 34 (-3 to 70) | 26 (-4 to 55) | 16 (-33 to 66) | 211 (88 to 333) |
| **Guatemala** | 5 151 (770 to 9532) | 1 734 (-45 to 3512) | 1 556 (-81 to 3192) | 1 124 (-2095 to 4343) | 9 565 (4315 to 14816) |
| **Guinea** | 2 523 (307 to 4739) | 648 (-101 to 1396) | 456 (-174 to 1086) | 239 (-765 to 1242) | 3 866 (1583 to 6149) |
| **Guinea Bissau** | 251 (-1 to 503) | 63 (-22 to 148) | 45 (-30 to 120) | 23 (-99 to 145) | 382 (125 to 639) |
| **Guyana** | 313 (69 to 557) | 91 (6 to 176) | 74 (4 to 144) | 50 (-87 to 188) | 528 (254 to 802) |
| **Haiti** | 3 164 (456 to 5873) | 704 (9 to 1399) | 538 (-13 to 1090) | 356 (-712 to 1425) | 4 763 (1905 to 7622) |
| **Honduras** | 2 892 (143 to 5640) | 994 (-144 to 2131) | 893 (-157 to 1942) | 668 (-1284 to 2621) | 5 446 (2258 to 8635) |
| **Hungary** | 23 617 (2937 to 44297) | 6 890 (-352 to 14132) | 5 131 (-746 to 11009) | 2 752 (-6296 to 11801) | 38 391 (17056 to 59725) |
| **Iceland** | 237 (50 to 424) | 132 (15 to 248) | 152 (32 to 272) | 128 (-178 to 434) | 649 (287 to 1011) |
| **India** | 329 410 (73528 to 585291) | 85 050 (5199 to 164901) | 68 824 (-241 to 137889) | 55 359 (-96178 to 206897) | 538 643 (243493 to 833793) |
| **Indonesia** | 119 488 (31067 to 207909) | 28 026 (3020 to 53033) | 19 867 (1104 to 38631) | 12 305 (-22698 to 47308) | 179 686 (85343 to 274030) |
| **Iran** | 32 132 (13508 to 50757) | 16 245 (4610 to 27879) | 16 023 (6151 to 25895) | 10 623 (-14736 to 35981) | 75 023 (40882 to 109163) |
| **Iraq** | 21 886 (5023 to 38748) | 5 525 (427 to 10623) | 3 793 (12 to 7573) | 1 916 (-4244 to 8075) | 33 119 (15604 to 50635) |
| **Ireland** | 4 129 (330 to 7928) | 1 481 (-62 to 3023) | 1 458 (-23 to 2938) | 1 153 (-1975 to 4282) | 8 221 (3469 to 12973) |
| **Israel** | 5 659 (1563 to 9756) | 2 688 (421 to 4954) | 2 793 (647 to 4939) | 2 194 (-3137 to 7526) | 13 334 (6444 to 20224) |
| **Italy** | 116 454 (43239 to 189670) | 53 083 (11937 to 94228) | 52 343 (16886 to 87799) | 39 200 (-53548 to 131948) | 261 080 (137731 to 384428) |
| **Jamaica** | 2 350 (338 to 4363) | 603 (-41 to 1247) | 450 (-44 to 943) | 284 (-535 to 1102) | 3 687 (1618 to 5755) |
| **Japan** | 296 010 (120231 to 471788) | 123 686 (32020 to 215351) | 121 141 (43220 to 199061) | 93 876 (-128124 to 315877) | 634 712 (335823 to 933602) |
| **Jordan** | 3 736 (1361 to 6111) | 1 370 (327 to 2412) | 1 109 (324 to 1895) | 625 (-956 to 2205) | 6 840 (3861 to 9818) |
| **Kazakhstan** | 12 543 (3981 to 21106) | 3 988 (805 to 7171) | 3 068 (683 to 5454) | 1 723 (-2853 to 6300) | 21 323 (11449 to 31198) |
| **Kenya** | 7 944 (1092 to 14796) | 2 119 (-84 to 4322) | 1 689 (-266 to 3643) | 1 059 (-2666 to 4783) | 12 810 (5257 to 20363) |
| **Kiribati** | 28 (-2 to 58) | 5 (-2 to 12) | 4 (-2 to 10) | 2 (-8 to 13) | 40 (9 to 70) |
| **Kuwait** | 1 824 (493 to 3156) | 623 (83 to 1163) | 511 (66 to 957) | 303 (-533 to 1139) | 3 262 (1692 to 4832) |
| **Kyrgyzstan** | 3 273 (830 to 5715) | 1 024 (114 to 1933) | 780 (56 to 1504) | 438 (-821 to 1698) | 5 515 (2808 to 8222) |
| **Lao PDR** | 1 634 (156 to 3113) | 441 (-95 to 976) | 339 (-157 to 836) | 204 (-699 to 1106) | 2 618 (980 to 4255) |
| **Latvia** | 4 613 (977 to 8249) | 1 475 (43 to 2908) | 1 110 (-54 to 2273) | 541 (-1249 to 2331) | 7 739 (3846 to 11632) |
| **Lebanon** | 4 580 (1145 to 8015) | 1 466 (199 to 2732) | 1 184 (132 to 2236) | 683 (-1265 to 2631) | 7 913 (3991 to 11834) |
| **Lesotho** | 629 (122 to 1136) | 157 (1 to 313) | 115 (-15 to 245) | 71 (-159 to 301) | 972 (433 to 1511) |
| **Liberia** | 819 (109 to 1530) | 201 (-29 to 431) | 142 (-55 to 339) | 72 (-252 to 396) | 1 235 (495 to 1975) |
| **Libya** | 3 338 (763 to 5913) | 1 152 (88 to 2216) | 892 (35 to 1750) | 497 (-931 to 1926) | 5 879 (3042 to 8716) |
| **Lithuania** | 7 418 (1696 to 13140) | 2 171 (107 to 4235) | 1 526 (-100 to 3153) | 686 (-1712 to 3084) | 11 801 (5836 to 17767) |
| **Luxembourg** | 437 (136 to 738) | 199 (37 to 362) | 214 (60 to 369) | 180 (-251 to 610) | 1 031 (493 to 1568) |
| **Macedonia (TFYR)** | 3 449 (474 to 6425) | 1 074 (-44 to 2193) | 814 (-117 to 1744) | 438 (-1009 to 1885) | 5 775 (2667 to 8883) |
| **Madagascar** | 3 803 (-365 to 7972) | 878 (-414 to 2169) | 672 (-483 to 1827) | 404 (-1509 to 2317) | 5 757 (1506 to 10008) |
| **Malawi** | 2 840 (733 to 4947) | 1 040 (109 to 1970) | 954 (148 to 1760) | 737 (-1152 to 2626) | 5 570 (2743 to 8397) |
| **Malaysia** | 16 972 (4917 to 29027) | 4 743 (405 to 9081) | 3 446 (97 to 6795) | 2 039 (-3912 to 7989) | 27 200 (14034 to 40366) |
| **Maldives** | 165 (29 to 300) | 56 (-2 to 115) | 45 (-7 to 98) | 30 (-64 to 124) | 296 (141 to 452) |
| **Mali** | 3 222 (172 to 6272) | 866 (-236 to 1967) | 642 (-309 to 1594) | 344 (-1250 to 1938) | 5 074 (1892 to 8256) |
| **Malta** | 434 (117 to 750) | 211 (30 to 391) | 222 (46 to 397) | 178 (-261 to 618) | 1 044 (495 to 1594) |
| **Mauritania** | 930 (-14 to 1874) | 232 (-85 to 550) | 166 (-114 to 446) | 85 (-364 to 533) | 1 412 (452 to 2373) |
| **Mauritius** | 833 (254 to 1412) | 330 (56 to 605) | 300 (62 to 538) | 216 (-319 to 750) | 1 679 (879 to 2478) |
| **Mexico** | 50 021 (15464 to 84579) | 16 653 (3180 to 30127) | 14 994 (3621 to 26368) | 11 456 (-16731 to 39643) | 93 125 (47450 to 138799) |
| **Micronesia (Federated States of)** | 25 (3 to 46) | 7 (-0 to 15) | 6 (-1 to 14) | 5 (-9 to 19) | 43 (19 to 68) |
| **Moldova** | 6 483 (1567 to 11399) | 1 715 (84 to 3346) | 1 156 (-52 to 2364) | 496 (-1282 to 2274) | 9 850 (4781 to 14920) |
| **Mongolia** | 1 301 (375 to 2226) | 360 (66 to 653) | 268 (50 to 486) | 146 (-265 to 557) | 2 074 (1050 to 3099) |
| **Montenegro** | 1 009 (114 to 1903) | 313 (-21 to 647) | 237 (-39 to 513) | 127 (-279 to 533) | 1 685 (766 to 2605) |
| **Morocco** | 21 663 (5675 to 37650) | 7 267 (992 to 13543) | 5 948 (782 to 11114) | 3 503 (-6360 to 13366) | 38 381 (19553 to 57209) |
| **Mozambique** | 4 606 (275 to 8936) | 1 002 (-168 to 2172) | 744 (-270 to 1758) | 434 (-1367 to 2234) | 6 786 (2280 to 11291) |
| **Myanmar** | 25 473 (3915 to 47031) | 5 652 (-596 to 11899) | 3 913 (-1345 to 9170) | 2 085 (-6728 to 10898) | 37 122 (14807 to 59437) |
| **Namibia** | 947 (40 to 1853) | 188 (-51 to 427) | 131 (-73 to 335) | 73 (-267 to 413) | 1 339 (416 to 2261) |
| **Nepal** | 8 743 (1527 to 15959) | 1 668 (117 to 3220) | 1 122 (-64 to 2307) | 680 (-1583 to 2943) | 12 213 (4675 to 19751) |
| **Netherlands** | 18 460 (4947 to 31973) | 8 539 (1332 to 15745) | 9 194 (2309 to 16079) | 7 631 (-10518 to 25779) | 43 823 (20783 to 66864) |
| **New Zealand** | 4 861 (1197 to 8526) | 1 785 (271 to 3299) | 1 785 (395 to 3174) | 1 423 (-2083 to 4929) | 9 854 (4696 to 15011) |
| **Nicaragua** | 2 789 (46 to 5531) | 828 (-200 to 1857) | 700 (-236 to 1636) | 500 (-1130 to 2130) | 4 817 (1818 to 7817) |
| **Niger** | 3 177 (100 to 6254) | 680 (-225 to 1585) | 455 (-321 to 1232) | 204 (-1033 to 1441) | 4 516 (1398 to 7634) |
| **Nigeria** | 32 904 (9362 to 56446) | 8 491 (976 to 16006) | 5 857 (175 to 11540) | 2 622 (-6921 to 12165) | 49 874 (24588 to 75160) |
| **North Korea** | 13 566 (776 to 26356) | 4 890 (-875 to 10655) | 4 385 (-1340 to 10110) | 3 161 (-7542 to 13864) | 26 002 (10441 to 41563) |
| **Norway** | 5 104 (820 to 9389) | 2 286 (84 to 4489) | 2 432 (234 to 4630) | 1 992 (-2997 to 6981) | 11 815 (5350 to 18279) |
| **Occupied Palestinian Territory** | 310 469 (69568 to 551370) | 109 216 (7312 to 211119) | 87 720 (2918 to 172522) | 50 835 (-95888 to 197557) | 558 239 (282986 to 833493) |
| **Oman** | 1 389 (350 to 2427) | 390 (48 to 733) | 292 (28 to 556) | 155 (-303 to 614) | 2 226 (1097 to 3356) |
| **Pakistan** | 45 215 (178 to 90251) | 7 382 (-3737 to 18502) | 4 786 (-4522 to 14095) | 2 884 (-12743 to 18510) | 60 267 (14825 to 105709) |
| **Panama** | 2 534 (242 to 4826) | 795 (-91 to 1682) | 689 (-100 to 1478) | 501 (-948 to 1949) | 4 519 (1924 to 7114) |
| **Papua New Guinea** | 1 027 (-149 to 2202) | 293 (-136 to 722) | 250 (-173 to 674) | 173 (-578 to 925) | 1 743 (449 to 3037) |
| **Paraguay** | 5 689 (721 to 10657) | 1 207 (-127 to 2540) | 850 (-154 to 1854) | 522 (-1062 to 2106) | 8 268 (3257 to 13279) |
| **Peru** | 9 119 (2930 to 15307) | 3 759 (832 to 6687) | 4 180 (1336 to 7025) | 4 347 (-5983 to 14676) | 21 405 (9069 to 33741) |
| **Philippines** | 31 938 (6568 to 57308) | 8 047 (-160 to 16255) | 5 836 (-1128 to 12801) | 3 147 (-8621 to 14915) | 48 969 (21884 to 76054) |
| **Poland** | 87 093 (27488 to 146699) | 25 462 (5306 to 45617) | 18 710 (4590 to 32829) | 9 744 (-15972 to 35459) | 141 008 (75197 to 206820) |
| **Portugal** | 15 174 (4412 to 25935) | 6 980 (1116 to 12844) | 6 902 (1513 to 12291) | 5 186 (-7508 to 17880) | 34 241 (17318 to 51164) |
| **Puerto Rico** | 6 032 (3 to 12061) | 1 621 (-430 to 3671) | 1 276 (-489 to 3041) | 847 (-1994 to 3688) | 9 776 (3604 to 15947) |
| **Qatar** | 441 (104 to 779) | 157 (16 to 298) | 126 (10 to 242) | 73 (-132 to 278) | 798 (411 to 1185) |
| **Romania** | 45 840 (13860 to 77820) | 12 833 (2044 to 23622) | 9 101 (1398 to 16804) | 4 591 (-8183 to 17365) | 72 366 (38022 to 106709) |
| **Russian Federation** | 242 057 (83188 to 400927) | 75 619 (14065 to 137172) | 53 981 (10686 to 97277) | 24 078 (-46856 to 95012) | 395 736 (218851 to 572620) |
| **Rwanda** | 1 993 (3 to 3984) | 553 (-144 to 1250) | 461 (-194 to 1115) | 300 (-905 to 1505) | 3 308 (1119 to 5497) |
| **Saint Lucia** | 133 (18 to 248) | 39 (-2 to 80) | 32 (-3 to 66) | 21 (-40 to 83) | 225 (100 to 350) |
| **Saint Vincent and the Grenadines** | 91 (20 to 162) | 29 (2 to 56) | 23 (1 to 46) | 16 (-27 to 58) | 159 (78 to 240) |
| **Samoa** | 19 (1 to 38) | 5 (-1 to 10) | 4 (-1 to 9) | 3 (-6 to 12) | 31 (11 to 51) |
| **Sao Tome and Principe** | 56 (13 to 98) | 12 (1 to 23) | 8 (-0 to 16) | 4 (-10 to 18) | 80 (35 to 124) |
| **Saudi Arabia** | 7 318 (1630 to 13006) | 2 681 (214 to 5147) | 2 310 (97 to 4523) | 1 398 (-2771 to 5566) | 13 707 (6727 to 20687) |
| **Senegal** | 3 495 (410 to 6580) | 846 (-158 to 1850) | 595 (-240 to 1430) | 316 (-1039 to 1672) | 5 253 (2058 to 8447) |
| **Serbia** | 16 184 (4894 to 27473) | 5 307 (832 to 9782) | 3 928 (537 to 7319) | 2 018 (-3471 to 7508) | 27 437 (14954 to 39920) |
| **Seychelles** | 73 (17 to 130) | 21 (1 to 41) | 16 (1 to 31) | 10 (-18 to 37) | 120 (59 to 182) |
| **Sierra Leone** | 1 561 (124 to 2997) | 341 (-94 to 776) | 228 (-144 to 600) | 103 (-492 to 698) | 2 233 (771 to 3694) |
| **Singapore** | 3 440 (1206 to 5673) | 1 641 (375 to 2907) | 1 695 (539 to 2852) | 1 386 (-1860 to 4632) | 8 162 (4061 to 12263) |
| **Slovakia** | 8 815 (2374 to 15256) | 3 083 (359 to 5808) | 2 417 (210 to 4624) | 1 347 (-2457 to 5150) | 15 662 (8336 to 22988) |
| **Slovenia** | 5 252 (611 to 9892) | 1 630 (-114 to 3374) | 1 235 (-192 to 2661) | 664 (-1517 to 2845) | 8 780 (3971 to 13589) |
| **Solomon Islands** | 107 (17 to 197) | 36 (-0 to 72) | 31 (-2 to 64) | 22 (-45 to 88) | 195 (87 to 303) |
| **Somalia** | 2 188 (52 to 4325) | 557 (-126 to 1239) | 436 (-181 to 1053) | 273 (-793 to 1340) | 3 455 (1197 to 5712) |
| **South Africa** | 30 070 (7652 to 52487) | 7 058 (1017 to 13099) | 5 123 (611 to 9636) | 3 261 (-5665 to 12188) | 45 512 (21397 to 69627) |
| **South Korea** | 36 449 (13615 to 59282) | 18 302 (4648 to 31957) | 20 924 (7724 to 34125) | 18 334 (-24324 to 60991) | 94 009 (43255 to 144763) |
| **South Sudan** | 1 330 (-125 to 2786) | 354 (-140 to 848) | 285 (-185 to 755) | 183 (-608 to 974) | 2 152 (622 to 3683) |
| **Spain** | 43 794 (14076 to 73512) | 24 519 (4666 to 44372) | 28 677 (8861 to 48494) | 24 261 (-32535 to 81058) | 121 252 (54830 to 187673) |
| **Sri Lanka** | 14 865 (2624 to 27106) | 3 915 (-191 to 8021) | 3 053 (-565 to 6672) | 2 227 (-4595 to 9050) | 24 060 (10497 to 37624) |
| **Sudan** | 13 016 (670 to 25362) | 2 639 (-454 to 5732) | 1 915 (-696 to 4527) | 1 074 (-3360 to 5508) | 18 644 (6013 to 31276) |
| **Suriname** | 366 (53 to 679) | 101 (-6 to 209) | 80 (-8 to 167) | 53 (-102 to 208) | 600 (265 to 936) |
| **Swaziland** | 301 (52 to 550) | 72 (-3 to 147) | 52 (-10 to 113) | 31 (-74 to 137) | 456 (196 to 716) |
| **Sweden** | 10 295 (3057 to 17534) | 4 670 (807 to 8534) | 4 938 (1252 to 8624) | 3 980 (-5633 to 13594) | 23 884 (11549 to 36219) |
| **Switzerland** | 5 802 (1309 to 10294) | 3 404 (419 to 6388) | 4 279 (946 to 7611) | 4 153 (-5728 to 14033) | 17 637 (6596 to 28677) |
| **Syrian Arab Republic** | 9 656 (1615 to 17697) | 3 134 (-38 to 6306) | 2 421 (-209 to 5051) | 1 356 (-2968 to 5680) | 16 567 (7860 to 25274) |
| **Taiwan** | 15 385 (3499 to 27270) | 7 096 (394 to 13797) | 7 391 (676 to 14107) | 6 267 (-10059 to 22594) | 36 139 (15956 to 56322) |
| **Tajikistan** | 3 752 (1118 to 6387) | 1 068 (197 to 1938) | 779 (126 to 1433) | 419 (-731 to 1569) | 6 019 (3128 to 8909) |
| **Tanzania** | 10 327 (1303 to 19350) | 2 702 (-166 to 5569) | 2 091 (-438 to 4620) | 1 133 (-3230 to 5496) | 16 252 (6663 to 25840) |
| **Thailand** | 54 959 (9031 to 100887) | 18 012 (-1742 to 37766) | 14 842 (-3585 to 33269) | 9 995 (-24239 to 44229) | 97 808 (43784 to 151832) |
| **Timor-Leste** | 406 (-14 to 826) | 74 (-35 to 182) | 47 (-52 to 146) | 18 (-153 to 189) | 545 (117 to 974) |
| **Togo** | 1 275 (86 to 2464) | 311 (-75 to 696) | 220 (-123 to 562) | 106 (-467 to 679) | 1 911 (675 to 3147) |
| **Tonga** | 54 (10 to 98) | 13 (1 to 26) | 10 (1 to 20) | 7 (-12 to 25) | 84 (37 to 131) |
| **Trinidad and Tobago** | 1 181 (122 to 2240) | 313 (-35 to 660) | 248 (-42 to 538) | 168 (-341 to 677) | 1 910 (794 to 3025) |
| **Tunisia** | 8 758 (2149 to 15367) | 3 293 (314 to 6273) | 2 782 (264 to 5301) | 1 706 (-2997 to 6408) | 16 540 (8559 to 24521) |
| **Turkey** | 70 255 (24520 to 115991) | 28 503 (5746 to 51260) | 24 371 (5923 to 42819) | 14 949 (-23577 to 53474) | 138 078 (75968 to 200188) |
| **Turkmenistan** | 2 399 (729 to 4068) | 760 (141 to 1378) | 600 (126 to 1074) | 346 (-618 to 1310) | 4 104 (2155 to 6054) |
| **Uganda** | 5 085 (-315 to 10484) | 1 096 (-386 to 2579) | 824 (-545 to 2193) | 424 (-1956 to 2804) | 7 429 (1805 to 13053) |
| **Ukraine** | 79 820 (27992 to 131648) | 25 224 (5196 to 45252) | 18 254 (4050 to 32457) | 8 604 (-15481 to 32688) | 131 902 (73658 to 190145) |
| **United Arab Emirates** | 1 206 (315 to 2097) | 370 (50 to 691) | 294 (35 to 553) | 168 (-317 to 653) | 2 038 (1023 to 3053) |
| **United Kingdom** | 54 189 (20247 to 88130) | 23 415 (6144 to 40685) | 25 297 (9507 to 41087) | 21 428 (-28782 to 71638) | 124 328 (60684 to 187971) |
| **United States of America** | 399 539 (121406 to 677673) | 153 386 (32181 to 274591) | 153 569 (47329 to 259810) | 121 595 (-167076 to 410266) | 828 090 (414185 to 1241995) |
| **Uruguay** | 5 302 (1294 to 9311) | 1 684 (154 to 3214) | 1 391 (138 to 2644) | 942 (-1506 to 3389) | 9 319 (4694 to 13944) |
| **Uzbekistan** | 13 633 (3161 to 24105) | 3 319 (494 to 6143) | 2 373 (339 to 4407) | 1 189 (-2491 to 4869) | 20 514 (9379 to 31649) |
| **Vanuatu** | 77 (3 to 151) | 19 (-4 to 43) | 15 (-6 to 36) | 10 (-25 to 44) | 121 (43 to 198) |
| **Venezuela** | 16 342 (3926 to 28757) | 4 983 (528 to 9437) | 4 188 (471 to 7905) | 3 050 (-4730 to 10830) | 28 563 (14036 to 43089) |
| **Vietnam** | 41 485 (10549 to 72421) | 14 618 (1165 to 28070) | 12 723 (501 to 24945) | 9 121 (-16546 to 34788) | 77 947 (38566 to 117328) |
| **Yemen** | 6 040 (73 to 12008) | 1 855 (-396 to 4107) | 1 515 (-565 to 3594) | 805 (-2813 to 4424) | 10 216 (3723 to 16708) |
| **Zambia** | 2 276 (413 to 4139) | 680 (25 to 1335) | 570 (-10 to 1149) | 380 (-782 to 1542) | 3 905 (1752 to 6059) |
| **Zimbabwe** | 3 321 (-96 to 6737) | 754 (-281 to 1789) | 543 (-350 to 1436) | 327 (-1086 to 1741) | 4 945 (1499 to 8391) |

**Supplementary Methods 1**

**List of international and governmental websites searched for high quality publicly available databases**

United Nations Population Prospectus

Global Burden of Disease

MIT Geoweb

World Pop

Data Finder: Population Reference Bureau

Integrated Public Use Microdata Series

World Bank

Centers for Disease Control and Prevention

United States Census Bureau

International Federation of Data Organisations (IFDO)

**Supplementary Methods 2**

**Search strategy to determine hazard ratios for age at time of initial hypertension diagnosis and dementia**

("hazard ratio"[Title/Abstract] OR "odds ratio"[Title/Abstract] OR "risk"[Title/Abstract] OR "associat*"[Title/Abstract])

AND

("hypertension"[Title/Abstract] OR "elevated blood pressure"[Title/Abstract])

AND

("dementia"[Title/Abstract] OR "cognit*"[Title/Abstract])

AND

("age at diagnosis"[Title/Abstract] OR "midlife"[Title/Abstract] OR "middle age*"[Title/Abstract] OR "late-life"[Title/Abstract])

There was no English language restriction. We did not find any relevant studies that were not in English in our search.

**Supplementary Methods 3**

**Statistical analysis**

Probabilities of incident hypertension in (2) are estimated by the difference in estimated prevalence of hypertension in successive age groups.

Country level, regional and global estimates of PAF are estimated using the formula:


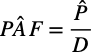
 (3)

where $\hat{P}$ is the estimated number of preventable dementia cases (corresponding to a specific country, a specific region or a global aggregation), and $D$represents estimated total dementia cases totaled over the same geographic area. $\hat{P}$ is estimated by summing country and gender specific estimated preventable cases:


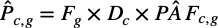
 (4)

where we assume that $F_{f}$=0.65 (that is 65% of dementia cases are female) and $F_{m}$=0.35, and $D_{c}$ are the estimated total dementia cases in country $c$. Total preventable cases can be partitioned into contributions by age group of incident hypertension using a similar formula:


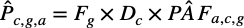
 (5)

The parametric bootstrap, assuming a normal distribution for estimated log-relative risks, estimated hypertension prevalence and the total number of dementia cases, with standard errors for these quantities derived from published confidence intervals, was applied to the estimation procedure to generate confidence intervals for PAF.

Our described example in the main paper of the PAF of hypertension for dementia in the 45-54 age group in Ireland (2.79%) differs slightly from our quoted figure in the tables (2.75% [-0.12% to 5.61%]). This table figure is derived from the full procedure of calculating PAF separately for males and females in each group (based on separate calculated incidences). The overall PAF within males, is then the summed PAF for males over all the age groups – and similarly for females.  We assumed that 65% of the dementia cases were female, and calculated the number of preventable female dementia cases by PAF_female*(estimated number of female dementia cases), and similarly the number of preventable male dementia cases by PAF_male*(estimated number of male dementia cases).  We then calculated the overall PAF in Ireland by the ratio of the sum of the number of preventable cases to the total observed cases.

Supplementary Figure 1 - Gender differences in Population Attributable Fractions of hypertension for dementia by region.


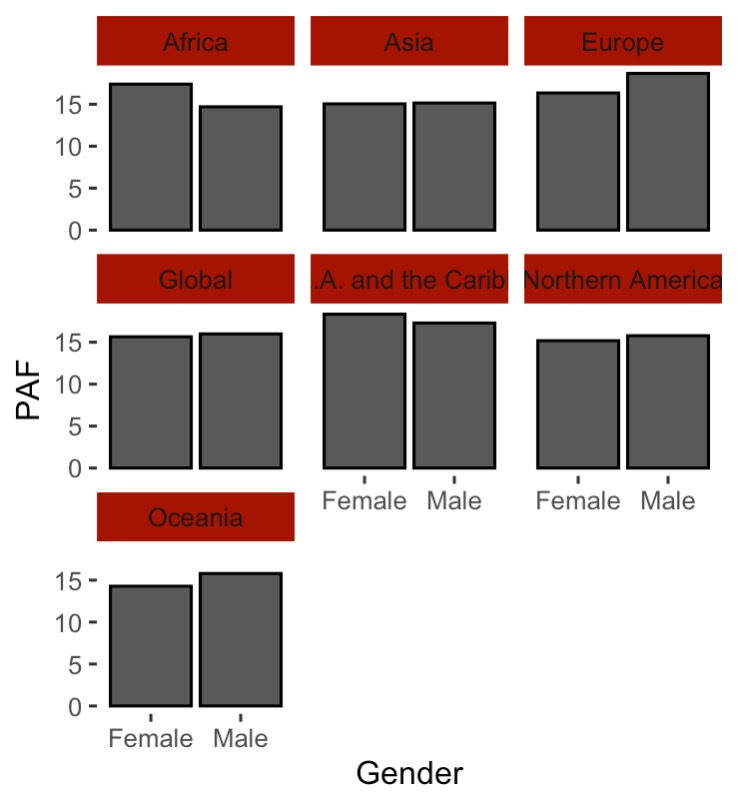


Supplementary Figure 2 - World map of hypertension prevalence by country (%, data extracted from NCD-Risc).


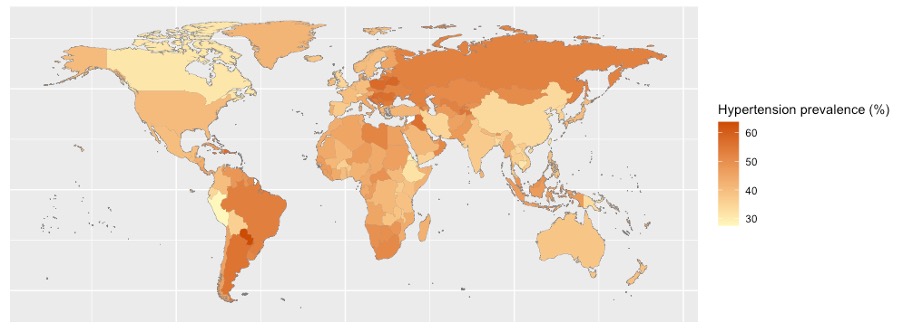


Supplementary Figure 3 - World map of dementia cases by country.


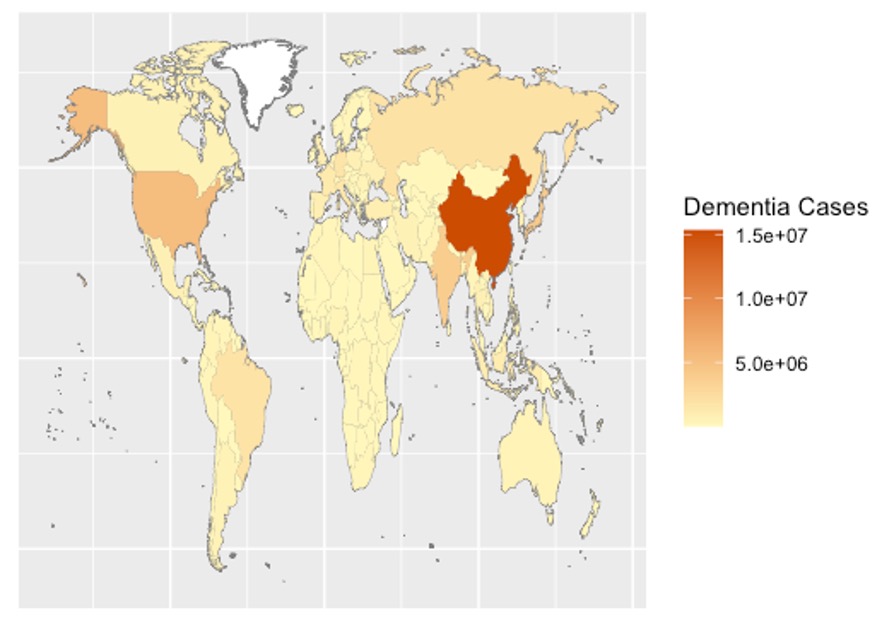


Supplementary Figure 4 - Number of dementia cases attributable to hypertension by region and age group at diagnosis.


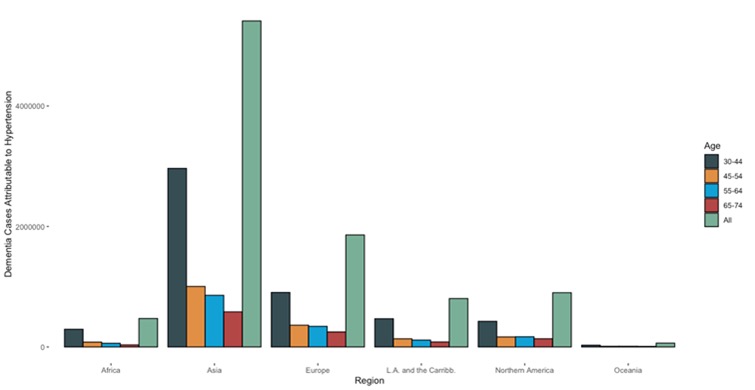

Supplement: Supplementary Methods 1–3, Supplementary Figs. S1–S4 and Supplementary Table S1–S6 [file mmc2.docx]
